# Genome-wide transcriptome profiling reveals functional networks involving the *Plasmodium falciparum* drug resistance transporters PfCRT and PfMDR1

**DOI:** 10.1186/s12864-015-2320-8

**Published:** 2015-12-21

**Authors:** Sophie H. Adjalley, Daniel Scanfeld, Elyse Kozlowski, Manuel Llinás, David A. Fidock

**Affiliations:** Department of Microbiology and Immunology, Columbia University Medical Center, New York, NY 10032 USA; Department of Molecular Biology & Lewis-Sigler Institute for Integrative Genomics, Princeton University, Princeton, NJ 08544 USA; Department of Biochemistry and Molecular Biology, Department of Chemistry, Center for Malaria Research and Center for Infectious Diseases Dynamics, Pennsylvania State University, University Park, PA 16802 USA; Division of Infectious Diseases, Department of Medicine, Columbia University Medical Center, New York, NY 10032 USA; Present addresses: Wellcome Trust Sanger Institute, Hinxton, UK; Present addresses: Google Inc., New York, NY 10011 USA; Present addresses: Pulmonary Center, Boston University School of Medicine, Boston, MA 02118 USA

**Keywords:** *Plasmodium falciparum*, Antimalarial drug resistance, Comparative transcriptomics, Transporters, Fast fourier transform, Gene set enrichment analysis, Hypergeometric analysis of time series

## Abstract

**Background:**

The acquisition of multidrug resistance by *Plasmodium falciparum* underscores the need to understand the underlying molecular mechanisms so as to counter their impact on malaria control. For the many antimalarials whose mode of action relates to inhibition of heme detoxification inside infected erythrocytes, the digestive vacuole transporters PfCRT and PfMDR1 constitute primary resistance determinants.

**Results:**

Using gene expression microarrays over the course of the parasite intra-erythrocytic developmental cycle, we compared the transcriptomic profiles between *P. falciparum* strains displaying mutant or wild-type *pfcrt* or varying in *pfcrt* or *pfmdr1* expression levels. To account for differences in the time of sampling, we developed a computational method termed Hypergeometric Analysis of Time Series, which combines Fast Fourier Transform with a modified Gene Set Enrichment Analysis. Our analysis revealed coordinated changes in genes involved in protein catabolism, translation initiation and DNA/RNA metabolism. We also observed differential expression of genes with a role in transport or coding for components of the digestive vacuole. Interestingly, a global comparison of all profiled transcriptomes uncovered a tight correlation between the transcript levels of *pfcrt* and *pfmdr1,* extending to dozens of other genes, suggesting an intricate regulatory balance in order to maintain optimal physiological processes.

**Conclusions:**

This study provides insight into the mechanisms by which *P. falciparum* adjusts to the acquisition of mutations or gene amplification in key transporter loci that mediate drug resistance. Our results implicate several biological pathways that may be differentially regulated to compensate for impaired transporter function and alterations in parasite vacuole physiology.

**Electronic supplementary material:**

The online version of this article (doi:10.1186/s12864-015-2320-8) contains supplementary material, which is available to authorized users.

## Background

With an estimated 214 million clinical cases and 438,000 deaths in 2015 [1] malaria remains one of the deadliest infectious diseases. Clinical manifestations are due to successive rounds of *Plasmodium* parasite invasion, replication within host erythrocytes, egress and re-invasion. Present efforts to reduce mortality and morbidity focus on the use of curative and preventive drug treatments as well as on vector control strategies [2]. Those include first-line artemisinin-based combination therapies (ACTs) that pair a highly potent artemisinin derivative with a longer-acting partner drug [3]. ACTs currently constitute the best regimens to treat 4-aminoquinoline- and antifolate-resistant *Plasmodium falciparum* parasites that have swept across the world. While 20 years were necessary for chloroquine (CQ) resistance to spread from its origins in Southeast Asia to Africa [4], resistance to the antifolate combination of sulfadoxine-pyrimethamine disseminated at a much faster pace [5]. The recent emergence of delayed parasite clearance following artesunate or ACT administration in Western Cambodia and Thailand [6–8] reemphasizes the need to develop therapeutic strategies to treat drug-resistant *P. falciparum* malaria and define the molecular basis of resistance.

Two key genetic determinants of *P. falciparum* resistance to antimalarial compounds are *pfcrt* [9] and *pfmdr1* [10, 11], which encode transporter proteins that localize to the digestive vacuole membrane of intra-erythrocytic parasites. While their native biological functions remain unknown, the inability to genetically disrupt *pfcrt* or *pfmdr1* provides evidence for their essentiality [12, 13]. Laboratory and field-based studies have demonstrated that PfCRT mutations constitute the primary determinant of in vitro CQ resistance and are associated with a substantially increased risk of CQ treatment failure in patients [14–16]. Studies with transgenic parasites or heterologous expression systems have attributed PfCRT-mediated CQ resistance to drug efflux out of the low-pH digestive vacuole, where CQ becomes diprotonated, concentrates up to 1,000-fold and acts by binding to heme products and preventing heme detoxification [17–20]. PfCRT mutations can also mediate cross-resistance to amodiaquine and increase susceptibility to lumefantrine, mefloquine and artesunate [15, 21, 22]. *pfmdr1* mutations as well as copy number variations have also been shown to impact *P. falciparum* susceptibility to several antimalarials [13, 22–26] presumably via drug transport across the digestive vacuole [27].

For PfCRT and PfMDR1, acquired resistance mutations, or in the case of *pfmdr1* gene amplifications, are thought to generally impart a fitness cost [28–31], presumably via altering their endogenous transport properties to the detriment of normal parasite development and replication. In endemic settings, this fitness cost manifests as a reduced prevalence of mutant *pfcrt* or *pfmdr1* following the release of selective drug pressure [32–36]. However, additional mutations elsewhere in an organism can compensate for the cost of acquiring resistance [37, 38] and restore fitness, as shown for *P. falciparum* resistance to antifolates [39–41]. Furthermore, the long-term propagation of drug-resistant strains even in the absence of selective pressure suggests adaptive mechanisms that counteract the detrimental effects of resistance-conferring genetic changes [42].

Intriguingly, the question of how parasites transcriptionally adjust in the long-term to the acquisition of mutations or gene amplifications in drug resistance transporters has scarcely been addressed. An earlier comparison of the transcriptional profiles of *pfcrt* mutant strains generated by CQ selection from a drug-sensitive parasite line suggested a limited number of changes in the parasite transcriptome [43]. That study, however, was restricted to a single time point of *P. falciparum* development within the host erythrocytes and as such it could not correct for temporal differences in the intra-erythrocytic developmental cycle (IDC) between parasite lines. Indeed, while transcriptome studies have suggested an inability of the parasite to transcriptionally respond to short-term drug pressure [44, 45], conceivably parasites may be able to evolve over multiple generations to states of optimal growth that adjust for physiological impacts of altered sequence or expression of drug/solute transporters. This is a testable hypothesis for PfCRT and PfMDR1, whose expression kinetics are closely tied to the formation of the parasite digestive vacuole during the trophozoite stage. Changes in the transcriptome profile of these mutant lines can be assessed in comparison with the carefully documented timeline of tightly regulated gene expression patterns that characterize the *P. falciparum* IDC [46–48]. These expression patterns underpin the biological processes of intracellular parasite development, replication and egress with accompanying morphological changes as parasites progress from rings to trophozoites to schizonts.

Here, we investigated how parasites adjusted transcriptionally to genetic modification of *pfcrt* or *pfmdr1,* resulting in altered drug resistance phenotypes following long-term culture. Using DNA microarrays, we conducted a genome-wide transcriptional profiling of genetically modified isogenic *P. falciparum* strains with either distinct PfCRT haplotypes or *pfmdr1* functional copy numbers throughout their 48-hour IDC. These previously engineered lines either display a loss of CQ resistance following the removal of the K76T mutation [20] (used epidemiologically as the primary molecular marker of CQ resistance) or show increased susceptibility to multiple antimalarial drugs (including mefloquine, lumefantrine, quinine and artemisinin) following genetic disruption of one of the two *pfmdr1* copies present in the multidrug resistant FCB line [26]. To analyze the data we developed a computational method that combines Fast Fourier Transform (FFT) and Gene Set Enrichment Analysis (GSEA) to correct for temporal differences between samples and compare microarray data between time series. We also used a reference pool of 11 profiled parasite lines to distinguish between stochastically variant genes and gene expression differences of biological significance, including those of subtle amplitude. Our results revealed a striking and previously unreported correlation between the expression levels of *pfcrt* and *pfmdr1*, and identified several pathways whose regulation was altered in response to changes in either gene, reinforcing the hypothesis of their functional interplay and the existence of coordinated functional networks.

## Results

### A Fast Fourier Transform-based method to align microarray time series data between profiled isogenic parasite cell lines

To assess the genomic impact of *P. falciparum* acquiring genetic changes in transporters that mediate antimalarial drug resistance, we profiled the transcriptome of multiple isogenic lines characterized by altered *pfcrt* and *pfmdr1* gene expression levels and/or corresponding protein haplotypes (Table 1). We also transcriptionally profiled the parental strains and performed both gene-specific and combined comparative analyses of the data sets.

**Table 1:** Summary of the *P. falciparum* lines used in the study. Drug resistance phenotype and *pfcrt*/*pfmdr1* phenotypes are indicated

					PfCRT haplotype^e^									PfMDR1 haplotype^e^					CN
Parasite Line	Parent	Rec.	Drug Phenotype	Genetic characteristic	72	74	75	76	220	271	326	356	371	86	184	1034	1042	1246	
FCB	--	No	Multi-drug resistant^a^	2 *pfmdr1* copies	**C**	**I**	**E**	**T**	**S**	**E**	**S**	I	**I**	N	Y	S	N	D	2
FCB^*pfmdr1_3′KD*^	FCB	Yes	CQ-resistant^b^	1 *pfmdr1* copy	**C**	**I**	**E**	**T**	**S**	**E**	**S**	I	**I**	N	Y	S	N	D	1
106/1	--	No	CQ-sensitive	2 *pfmdr1* copies, *pfcrt* K76	**C**	**I**	**E**	K	**S**	**E**	**S**	I	**I**	N	Y	S	N	D	2^f^
7G8	--	No	CQ-resistant	*pfcrt* K76T	**S**	M	N	**T**	**S**	**Q**	**D**	**L**	R	N	**F**	**C**	**D**	**Y**	1
7G8^*pfcrt_CTL*^	7G8	Yes	Less CQ-resistant^c^	*pfcrt* shorter 5′UTR, T76	**S**	M	N	**T**	**S**	**Q**	**D**	**L**	R	N	**F**	**C**	**D**	**Y**	1
7G8^*pfcrt_T76K*^	7G8	Yes	CQ-sensitive^d^	*pfcrt* shorter 5′UTR, K76	**S**	M	N	K	**S**	**Q**	**D**	**L**	R	N	**F**	**C**	**D**	**Y**	1

*CQ*: chloroquine,*CN: pfmdr1* copy number, *CTL:* control, *KD:* knockdown, *Rec:* Recombinant, *UTR:* Untranslated region

^a^Resistant to chloroquine through *pfcrt* mutations and partially resistant to lumefantrine, mefloquine and quinine through *pfmdr1* gene amplification

^b^Genetic ablation of 2nd *pfmdr1* copy number led to increased susceptibility to lumefantrine, mefloquine and quinine

^c^Reduced *pfcrt* expression through replacement of full-length 5′UTR with shorter version resulted in reduced level of CQ resistance

^d^Reversion of PfCRT K76T to wild-type K76 led to loss of CQ resistance

^e^Amino acids that differ from wild-type haplotypes (reference sequence 3D7, not shown) are shown in bold

^f^Transcriptome and qRT-PCR studies indicated that 106/1 expresses the equivalent of a single *pfmdr1* copy, despite having two physical copies

The first data set (focusing on *pfmdr1*) included FCB^*pfmdr1_3′KD*^, which was engineered via single-site crossover-based disruption of one of the two *pfmdr1* copies within the duplicated 100-kb genomic region of the multidrug-resistant parasite strain FCB [26, 49]. This resulted in a 50 % decrease in *pfmdr1* gene expression and a reduction of approximately 40 % in its protein level (Additional file 1: Figure S1A). In addition to FCB^*pfmdr1_3′KD*^ and its parental line FCB, our transcriptome analysis included 106/1, a parasite strain that genetically is nearly identical to FCB [50]. FCB and FCB^*pfmdr1_3′KD*^ display the same PfCRT haplotype (including the K76T mutation that is required for CQ resistance), whereas 106/1 has a key difference at position 76 that expresses a lysine (K) and confers CQ susceptibility [9, 51].

The second data set (*pfcrt*) included 7G8^*pfcrt_T76K*^ and 7G8^*pfcrt_CTL*^ parasite lines that were generated by allelic exchange via single crossover recombination targeting codon 76 of the mutant *pfcrt* allele in the CQ-resistant strain 7G8 [20]. The encoded threonine residue was back mutated to the wild-type lysine in 7G8^*pfcrt_T76K*^, resulting in a complete loss of CQ resistance, whereas it remained identical to 7G8 in the recombinant control 7G8^*pfcrt_CTL*^. Additionally, the two recombinant lines are characterized by a two-fold reduction in *pfcrt* mRNA and protein expression, allowing us to assess the impact of both the acquisition/loss of a point mutation and alteration in gene expression (Additional file 1: Figure S1B). Of note, 7G8^*pfcrt_CTL*^ is only moderately CQ-resistant, with a mean CQ IC_50_ value (109 nM) that is close to half that of the parental 7G8 line (251 nM) by virtue of the reduced PfCRT expression [20].

**Fig. 1:**
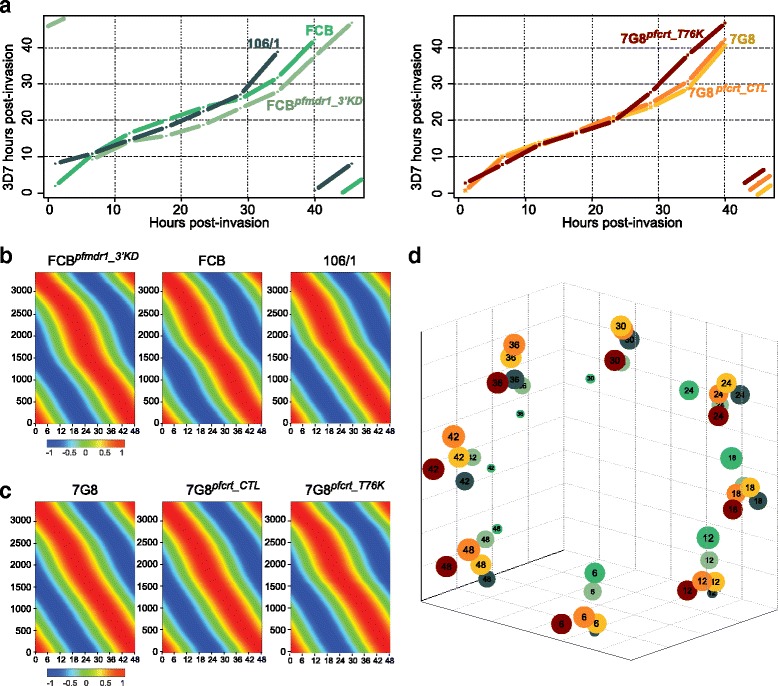
Alignment of all time series to the 3D7 reference transcriptome reveal differences in growth rates between the profiled parasite lines. **a** Plot of all time courses before temporal alignment of the transcriptome data. For each transcriptome data set, the original 8 time points of each 48-hour time course (corresponding to a single intra-erythrocytic developmental cycle (IDC)) were aligned to the transcriptome of 3D7 that earlier had been elucidated at a one-hour resolution [46]. **b** Phaseograms of all parasite lines in the FCB set after dynamic time warping. Genes (on the Y-axis) were ordered according to their phase of expression computed using Fast Fourier Transform (FFT), which interpolated expression levels at unassayed time points. The resulting output generated a smooth sine curve with a single peak of maximum expression (shown in red, spanning a heatpmap gradient to the minimum transcript levels shown in blue) for each gene across the entire 48-hour IDC. **c** Phaseograms of the three parasite lines in the 7G8 set after dynamic time warping. **d** Principal Component Analysis (PCA) confirming the proper alignment of all transcriptomes. The ~3,000 to ~3,500 genes represented in each transcriptome were used to generate a matrix of values that was reduced to a tri-dimensional projection using PCA. The first two principal components accurately recover the progression of the lines through the 48-hour lifecycle while the third principal component focuses on differences between the lines as symbolized by the size of the circles. Circle colors refer to the parasite lines depicted in (**a**)

To conduct our comparative analysis, tightly synchronous parasites were cultured for the length of a complete IDC (~48 h). RNA was extracted from samples collected every 6 h for 48 h and assayed on a 70-mer DNA microarray, representing more than 5,350 *P. falciparum* genes [52]. Alignment of each profiled time series to the transcriptome of the reference strain 3D7 showed small but significant differences in the IDC duration. Indeed, the transgenic strains displayed a shorter life cycle than their respective parents (Fig. 1a).

Given the resulting slight time shifts between the time series, we developed a computational method to align the microarray expression data and to ensure that our comparative analysis would not be skewed by temporal differences in the developmental stage of the different parasite lines at the time of harvesting. We used FFT to impute gene expression levels at 376 unassayed time points to obtain a 7.5-minute resolution of each time course (384 time points in total; see Methods, Additional file 2: Table S1). To validate this approach, we first performed a simulation using published transcriptome data sets that constituted of hourly time course data for the strains HB3, Dd2 and 3D7 [48]. For each strain we selected 8 time points spread 6 h apart, from which we generated a curve of 376 imputed points. Using Pearson correlation values for gene expression, we then compared each actual hourly time point to the imputed curve to identify the closest matching time point. We observed that actual time points from the original time courses matched imputed time points that were fairly evenly spread along the imputation-derived curve, with correlations of average 83–91 % across the range of time points for each strain. This suggests that the imputation method can work on datasets that comprise only eight time points, such as ours.

Using our imputed data sets, we then applied a dynamic time warping/Pearson Correlation Coefficient (PCC) method to determine for each profiled parasite line the optimal post-invasion time point (corresponding to the first sampling time point) along the IDC (see Methods). All time courses were then realigned to the 3D7 transcriptome and to each other when performing pairwise comparisons (Additional file 3: Figure S2).

Phaseograms representing the transcriptome of each strain were assembled by hierarchically clustering the mean-centered log_2_ expression ratios of the genes that passed the different filters (~3,500 genes), according to their phase of expression calculated as described above. Transcriptomes of all strains reproduced the cascade of expression typical of *P. falciparum*, in which genes are expressed in a highly periodic mode [46] (Fig. 1b, c). Furthermore, all profiled strains demonstrated excellent synchronicity, with tight clustering of the expression data as observed using Principal Component Analysis (PCA), thereby validating the alignment of the time series (Fig. 1d).

### Chromosomal mapping of gene expression changes reveals amplicon loss on chromosome 5 in FCB^*pfmdr1_3′KD*^ and down-regulation of a chromosome 2 gene cluster in 7G8^*pfcrt_T76K*^

To identify genes that were differentially expressed between parasite time series within each data set, steady state transcript levels were analyzed across the IDC for each profiled strain and compared to each other. We computed gene expression fold differences for each pairwise comparison as the difference between the areas under the curves, which were extracted from absolute temporal gene expression plots derived from the full 384 time point data.

**Fig. 2:**
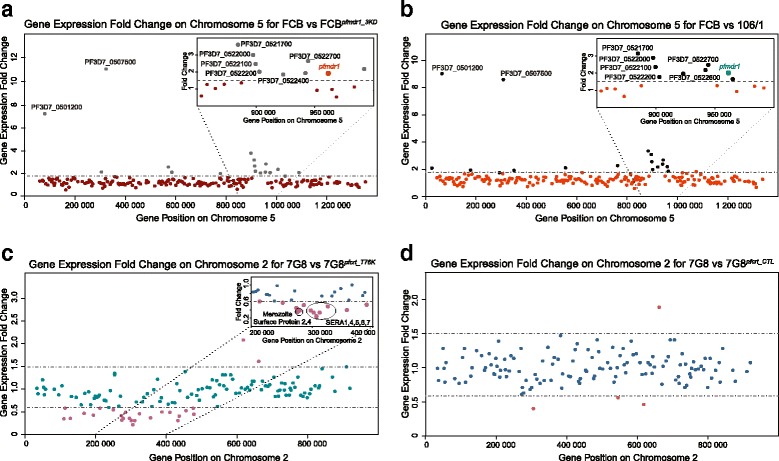
Differential gene expression detected by chromosome mapping. **a** Chromosome mapping of gene expression fold differences between FCB and FCB^*pfmdr1_3′KD*^ shows differential transcription of a large fragment on chromosome 5 corresponding to the *pfmdr1*-containing 100-kb amplicon region. For each gene located on chromosome 5, the expression fold difference between FCB and FCB^*pfmdr1_3′KD*^ was mapped using the chromosomal coordinates. Interestingly, PF3D7_0507500 (a subtilisin-like protease) and PF3D7_0501200 (a parasite-infected erythrocyte surface protein) show higher expression in FCB in comparison to both FCB^*pfmdr1_3′KD*^ and 106/1 (see panel B). The inset shows the genes within the *pfmdr1*-containing amplicon region. **b** Chromosome mapping of gene expression fold differences between FCB and 106/1 for each gene on chromosome 5 with the inset showing genes within the *pfmdr1*-containing amplicon region. **c** Chromosome mapping of gene expression fold differences between 7G8 and 7G8^*pfcrt_T76K*^ shows differential gene expression of a large number of genes located on chromosome 2 (inset shows a cluster of genes coding for proteins involved in parasite invasion). **d** Chromosome mapping of gene expression fold differences between 7G8 and 7G8^*pfcrt_CTL*^ for each gene on chromosome 2. In each pairwise comparison, the fold difference in gene expression was calculated as the difference between the areas under the curve computed on the full 384 time point data

We then generated chromosome maps in which fold changes in expression were plotted as a function of the chromosomal position of each gene. These maps revealed clusters of differential expression in telomeric regions of several chromosomes, possibly indicating hypervariable transcription of genes within these regions, as previously reported [53] (Additional file 4: Figure S3). Surprisingly, an entire set of genes all located on chromosome 5 and overlapping with the 100-kb amplicon containing *pfmdr1* (PF3D7_0523000) appeared to be differentially expressed between FCB and both FCB^*pfmdr1_3′KD*^ and 106/1 (Fig. 2a, b). All genes within this region except PF3D7_0521300, PF3D7_0521800, PF3D7_0522900, and PF3D7_0523100 were expressed two- to four-fold less in FCB^*pfmdr1_3′KD*^ and 106/1 as compared to FCB (Fig. 2a, b; Additional file 5: Table S2). Interestingly, global gene expression profiles within the amplicon region on chromosome 5 were extremely similar between FCB^*pfmdr1_3′KD*^ and 106/1 (Fig. 2a, b). Given the overlap with genes that constitute the amplicon in the FCB strain (Additional file 6: Figure S4), we performed quantitative real time PCR on parasite genomic DNA to assess whether the observed transcriptional differences between FCB and both FCB^*pfmdr1_3′KD*^ and 106/1 originated from different copy numbers within the *pfmdr1* region on chromosome 5. Quantitative PCR results indicated that the FCB^*pfmdr1_3′KD*^ strain has a single copy of the genes that are found duplicated in FCB, including *pfmdr1*. Furthermore, although our microarray data showed a similar down-regulation of these genes in 106/1, the parasite strain harbored two physical copies of these. These results with 106/1 indicate a different mechanism for reduced gene expression in that chromosomal region, potentially involving silencing of one of the two amplicons (Additional file 7: Table S3).

The same chromosomal mapping of gene expression fold changes revealed the down-regulation of a cluster of genes on chromosome 2 in the back-mutant strain 7G8^*pfcrt_T76K*^ in comparison to the background strain 7G8 (Fig. 2c; Additional file 8: Table S4). This down-regulation was specific to the back-mutant parasites, as no gene expression difference was observed in that region when comparing the transgenic control strain 7G8^*pfcrt_CTL*^ to 7G8 (Fig. 2d). Interestingly, many of the affected genes encode proteins involved in parasite invasion of host erythrocytes, including Merozoite Surface Proteins (PF3D7_0206800 and PF3D7_0207000) and Serine Repeat Antigen (SERA) gene products (PF3D7_0207400, PF3D7_0207500, PF3D7_0207600, PF3D7_0207700, and PF3D7_0207800) (Fig. 2c, Additional file 8: Table S4).

### Genetic or transcriptional deamplification of the *pfmdr1*-containing amplicon has broad transcriptional consequences

To assess the transcriptional variations between the profiled strains, we performed gene expression and gene set enrichment analyses, considering both absolute and normalized gene expression fold changes (Methods). We also conducted pairwise comparisons of the transcriptome data sets for three developmental stage windows, corresponding to rings (12 to 16 h post-invasion), trophozoites (24 to 28 h post-invasion) and schizonts (32 to 36 h post-invasion. These comparisons enabled us to detect changes in genes/gene sets within a short window of expression or genes/gene sets whose peak of expression occurred at different times between related parasite lines (Methods; Additional file 9: Figure S5). We observed that more than 70 % of all genes peaked within the same 8-hour window in the *pfmdr1* dataset while about 80 % of the genes peaked within the same 5-hour window in the *pfcrt* dataset (Additional file 10: Table S5). Overall, the great majority of the observed changes was driven by changes in gene expression levels as opposed to altered timing of expression.

**Fig. 3:**
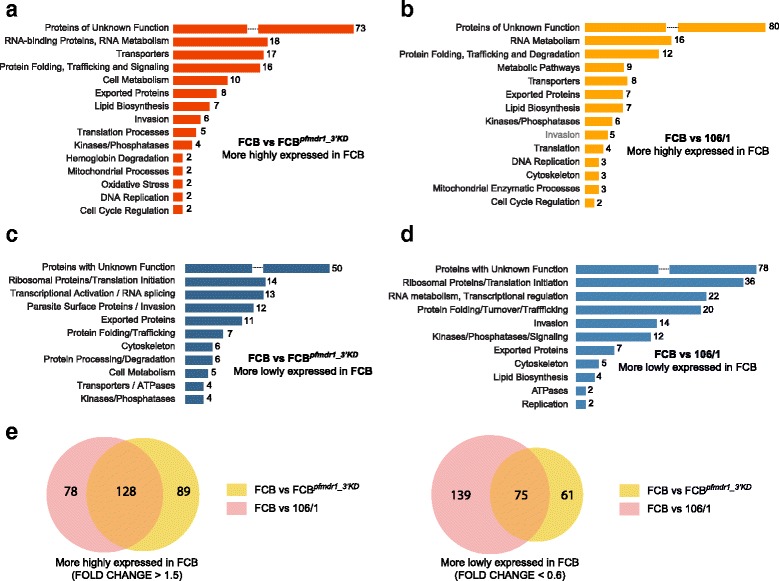
Genes with significant expression fold changes organized by categories for pairwise comparisons of the transcriptomes in the *pfmdr1* data set. Gene expression fold changes were calculated as the difference between the areas under the curve generated on the full 384 time point data and were then normalized using a baseline created by a background pool of 11 transcriptomes. For each gene, all 55 possible pairwise combinations of transcriptomes created a distribution of expression fold changes, which was used as a normalization factor. **a** Genes showing higher expression in the parental line FCB in comparison to *pfmdr1*-knock-down strain FCB^*pfmdr1_3′KD*^. To identify significant differences in gene expression, we applied a threshold of a fold change >1.5 and a normalized fold change at least 2 standard deviations (SD) above the mean. **b** Genes showing higher expression in FCB in comparison to 106/1. **c** Genes showing lower expression in the parental line FCB compared to the knock-down strain FCB^*pfmdr1_3′KD*^. We applied a threshold of a fold change <0.6 and a normalized fold change 3 SD below the mean. **d** Genes showing lower expression in FCB in comparison to 106/1. **e** Venn diagrams depicting the overlap of genes with higher (fold change >1.5, *left*) or lower (fold change <0.6, *right*) expression in FCB for the FCB vs. FCB^*pfmdr1_3′KD*^ and FCB vs. 106/1 pairwise comparisons

Analysis of the *pfmdr1* data set (FCB vs. FCB^*pfmdr1_3′KD*^ and FCB vs. 106/1) showed that many of the genes with significant differences in expression level encoded proteins of unknown function. This analysis also revealed decreased expression of multiple genes involved in RNA and protein metabolism upon deamplification of the *pfmdr1*-containing amplicon region on chromosome 5 (Fig. 3a, b). Among these were genes coding for RNA-binding proteins, splicing/exosome elements and protein chaperones and modifiers (Additional file 11: Table S6A,B). In contrast, numerous genes encoding ribosomal protein subunits, or involved in RNA translation initiation or protein turnover showed a more than two-fold higher expression level in both strains with lower *pfmdr1* expression (Fig. 3c, d). Furthermore, genes mainly involved in transcriptional control, including several elements of the basal transcription machinery, were 2 to 3.5-fold up-regulated in FCB^*pfmdr1_3′KD*^ and 106/1 in comparison to FCB. For instance, those encoding a putative RNA polymerase II (PF3D7_1027400), and the AP2-containing transcription factors PF3D7_0730300 and PF3D7_0613800 [54, 55] displayed higher expression in FCB^*pfmdr1_3′KD*^ (Additional file 11: Tables S6C). Genes encoding chromatin constituents such as the components of the nucleosome core histone H3 (PF3D7_0610400) and the histone variant H2B (PF3D7_0714000) were 2 to 2.5–fold less expressed in FCB in comparison to 106/1 (Additional file 11: Table S6D). These results suggest an alteration of the global transcriptional and translational activities in both strains with reduced *pfmdr1* expression in comparison to FCB.

**Fig. 4:**
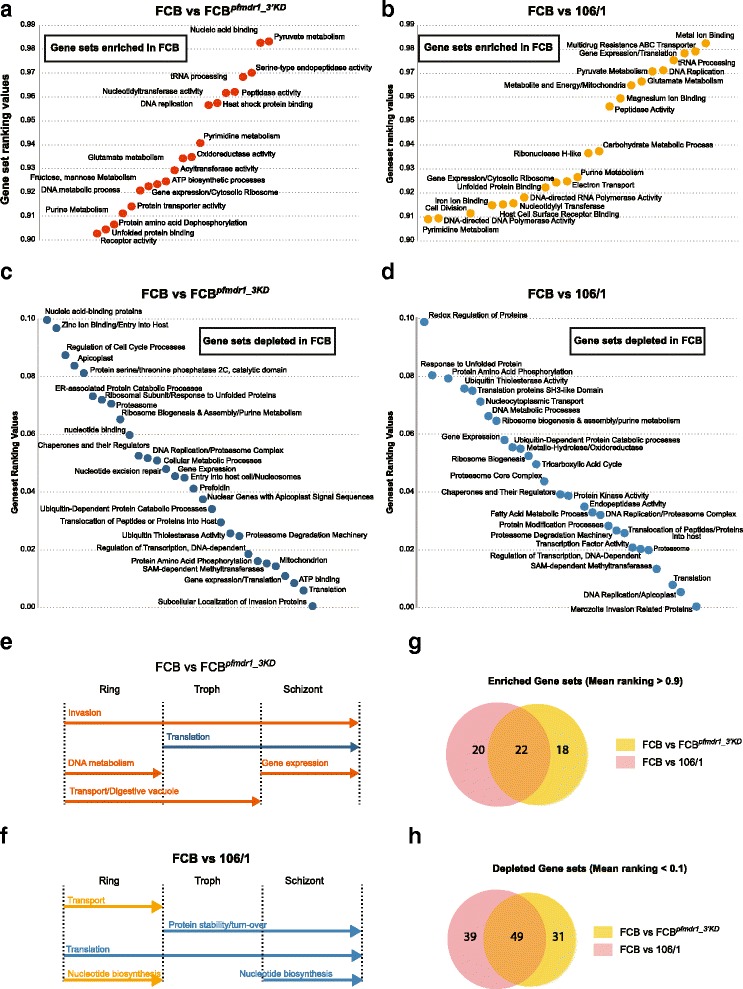
Hypergeometric Analysis of Time Series (HATS) for pairwise comparisons of the transcriptomes in the *pfmdr1* data set. HATS was performed by combining FFT to align the time series with a hypergeometric distribution approach based on random permutations to compute an enrichment score for each gene set. Normalization of the enrichment scores was performed using an expression baseline constituted by a background pool of 11 transcriptome data sets (see Methods). We applied a threshold of normalized gene set rank >0.9 for gene sets we considered positively enriched and normalized gene set rank <0.1 for gene sets considered negatively enriched. Gene sets were built using GO, KEGG and the Malaria Parasite Metabolic Pathway (listed in Additional file 26: Table S17). **a** Gene sets that are enriched in FCB in comparison to FCB^*pfmdr1_3′KD*^ (mean ranking values >0.9). **b** Gene sets that are enriched in FCB in comparison to 106/1 (mean ranking values >0.9). **c** Gene sets that are depleted in FCB in comparison to FCB^*pfmdr1_3′KD*^ (mean ranking values <0.1). **d** Gene sets that are depleted in FCB in comparison to 106/1 (mean ranking values <0.1). **e** Stage-associated enrichment analysis in FCB in comparison to FCB^*pfmdr1_3′KD*^. Gene sets with significant enrichment (orange) or depletion (blue) for three windows of the parasite IDC correspond to rings (R, 12–16 h post-invasion), trophozoites (T, 24–28 h post-invasion) and schizonts (S, 32–36 h post-invasion). **f** Stage-associated enrichment analysis in FCB in comparison to 106/1. Gene sets with significant enrichment (orange) or depletion (blue) during the same three stages as in (E). **g** Venn diagrams depicting overlaps of gene sets significantly enriched in FCB (mean rank of enrichment score >0.9) for both FCB vs. FCB^*pfmdr1_3′KD*^ and FCB vs. 106/1 pairwise comparisons, as identified from our HATS analysis. **h** Venn diagrams depicting overlaps of gene sets significantly depleted in FCB (mean rank of enrichment score <0.1) for both FCB vs. FCB^*pfmdr1_3′KD*^ and FCB vs. 106/1 pairwise comparisons, as identified using HATS

Multiple genes implicated in cellular metabolism, parasite remodeling and invasion also displayed higher transcript levels in FCB^*pfmdr1_3′KD*^ and 106/1 compared to FCB (Fig. 3c, d, Additional file 11: Table S6C,D). These observations were confirmed by applying a statistical approach that we termed Hypergeometric Analysis of Time Series (HATS), which combined Fast Fourier Transform and GSEA, to further assess the functional significance of the variations in gene expression observed between the parasite strains (see Methods). HATS, although based on GSEA, imputes a gene set enrichment score from the hypergeometric distribution of all gene sets calculated following 1,000 random permutations, instead of using a weighted Kolmogorov-Smirnov-like test [56]. HATS demonstrated that genes more highly expressed in FCB in comparison to FCB^*pfmdr1_3′KD*^ and 106/1 were enriched mainly for metabolic and enzymatic activities such as purine/pyrimidine and pyruvate/glutamate metabolic pathways (Fig. 4a, b). In contrast, several genes and gene sets associated with transcription regulation, translation initiation including ribosome biogenesis, and proteasome-mediated protein catabolism/turnover, were depleted in FCB in comparison to both strains with lower *pfmdr1* expression (Fig. 4c, d). Stage-enrichment analysis (Methods) revealed that these differences were detectable mainly during the trophozoite and schizont stages (Fig. 4e, f, Additional files 12 and 13: Tables S7 and S8 respectively). Some of these alterations, including those affecting protein synthesis and protein turnover via proteasome degradation have also been observed in the transcriptional profiles of artemisinin-resistant patient isolates carrying causal mutations in the kelch gene K13, suggesting that these pathways might contribute to general mechanisms adopted by multi-drug resistant parasites [57].

Further stage-specific analysis showed that gene categories comprising transporters or components localized to the parasite digestive vacuole were significantly enriched (>2-fold) in FCB in comparison to FCB^*pfmdr1_3′KD*^ in mature asexual blood stages, i.e. when the digestive vacuole is functioning. These genes included two V-type ATPases (PF3D7_1354400, PF3D7_0519200), an AAA family ATPase (PF3D7_0707400), an ABC transporter (PF3D7_0810200), and drug/metabolite and amino acid transporters (PF3D7_1428200 and PF3D7_1132500), (Fig. 4e, Additional file 12: Table S7A,C,G). These genes sometimes also displayed a higher expression in FCB in comparison to 106/1 (Fig. 4f, Additional file 13: Table S8A).

Overall, the global transcriptional response to the deamplification of the *pfmdr1*-containing amplicon was highly similar between FCB^*pfmdr1_3′KD*^ and 106/1. For instance, expression analysis of individual genes revealed that both FCB^*pfmdr1_3′KD*^ and 106/1 strains in comparison to FCB showed a 2- to 3-fold down-regulation of the genes coding for an ATP-dependent RNA helicase (PF3D7_0521700) and the H2 ribonuclease (PF3D7_0623900) (Additional file 11: Table S6E). These two genes were among the very few that were differentially expressed between the strains across all time points throughout the IDC, as demonstrated by stage-specific analysis (Additional files 12 and 13: Tables S7K and S8K respectively). HATS also identified several gene sets that were enriched or depleted in both FCB^*pfmdr1_3′KD*^ and 106/1 in comparison to FCB, which included parasite transporters, components of the proteasome degradation machinery and ribosome constituents (Additional file 11: Table S6G,H). Overall, we observed an overlap of greater than 50 % in the gene sets enriched or depleted in both FCB^*pfmdr1_3′KD*^ and 106/1 in comparison to FCB (Fig. 4g, h, Additional file 11: Table S6E,F). Interestingly, we also detected enrichment for gene sets associated with mitochondrial activities such as energy production and electron transport in the FCB versus 106/1 pairwise comparison, but not when comparing FCB with FCB^*pfmdr1_3′KD*^ (Fig. 4a, b). These gene sets in addition to those related to aminoacyl-tRNA biosynthesis, redox regulation or associated with the parasite digestive vacuole were differentially enriched between both *pfmdr1* knock-down strains (Additional file 14: Figure S6). The contrasting nature of the PfCRT haplotypes between the two strains might account for these differences. This aspect was further addressed when analyzing the *pfcrt* data set.

### Drug-resistance mutations in *pfcrt* affect protein synthesis gene expression

**Fig. 5:**
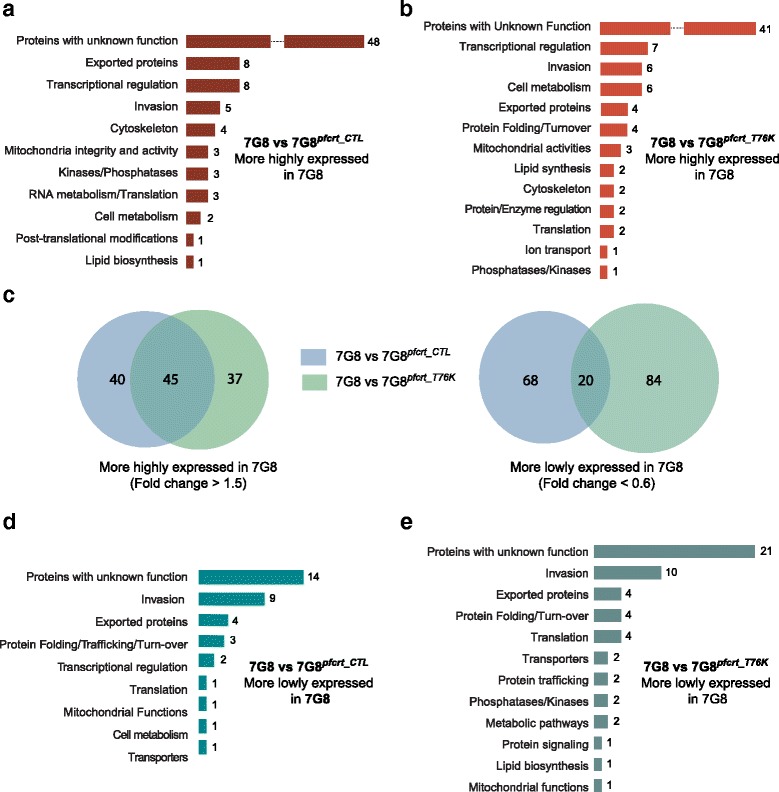
Genes with significant expression fold changes organized by categories for pairwise comparisons of the transcriptomes in the *pfcrt* data set. Gene expression fold changes were calculated as the difference between the areas under the curve generated on the full 384 time point data and were then normalized using a baseline created by the background pool of 11 transcriptomes. For each gene, all 55 possible pairwise combinations of transcriptomes created a distribution of expression fold changes, which was used as a normalization factor. To identify significant differences, we applied a threshold of a fold change >1.5 and a normalized fold change at least 2 standard deviations (SD) above the mean for higher gene expression, and a threshold of a Fold change <0.6 and a normalized fold change 3 SD below the mean for lower expression. **a** Genes with higher expression in the parental line 7G8 in comparison to the recombinant control strain 7G8^*pfcrt_CTL*^. **b** Genes showing higher expression in 7G8 compared to the recombinant back-mutant strain 7G8^*pfcrt_T76K*^. **c** Venn diagrams depicting the overlap of genes with higher (fold change >1.5, left) or lower (fold change <0.6, right) expression in 7G8 for both 7G8 vs. 7G8^*pfcrt_CTL*^ and 7G8 vs. 7G8^*pfcrt_T76K*^ pairwise comparisons. **d** Genes with lower expression in comparison with the recombinant control strain 7G8^*pfcrt_CTL*^. **e** Genes with lower expression in the parental line 7G8 in comparison with the recombinant back-mutant strain 7G8^*pfcrt_T76K*^

We applied analytical approaches similar to those described above to the *pfcrt* data set (Additional file 15: Table S9) and performed pairwise comparisons of 7G8, 7G8^*pfcrt_CTL*^ and 7G8^*pfcrt_T76K*^ transcriptomes. Expression analysis for individual genes showed that several genes encoding proteins involved in transcriptional regulation, such as histone deacetylases (PF3D7_1472200) and specific transcription factors (PF3D7_1222400, PF3D7_1222600) from the ApiAP2 family were globally less expressed in the transgenic back-mutant 7G8^*pfcrt_T76K*^ and control 7G8^*pfcrt_CTL*^ strains in comparison to the parental line 7G8 (Fig. 5a, b, Additional file 15: Table S9A,B). Additionally, genes involved in the maintenance of mitochondrial integrity and activity, such as the mitochondrial import inner membrane translocase subunits TIM13 and TIM44 (PF3D7_1242900 and PF3D7_1125400, respectively) and one of the cytochrome c1 precursors (PF3D7_1462700), showed 2- to 3.5-fold lower transcript levels in 7G8^*pfcrt_CTL*^ and 7G8^*pfcrt_T76K*^ in comparison to 7G8 (Fig. 5a, b, Additional file 15: Table S9A,B). Stage-specific analysis revealed that down-regulation of the genes encoding the TIM subunits was most apparent during the schizont stage for both transgenic strains (Additional files 16 and 17: Tables S10E and S11E respectively), which shared many of the genes displaying a decreased expression when compared to 7G8 (Fig. 5c, Additional file 15: Table S9C). On the other hand PF3D7_0609400, which encodes a cardiolipin synthase equally central to the mitochondrial inner membrane, was among the limited number of genes with higher transcript levels in both transgenic strains (Fig. 5c), particularly at the trophozoite stage (Additional files 16 and 17: Tables S10D and S11D). These observations suggest that decreased *pfcrt* expression, regardless of its haplotype in the 7G8 background, might affect mitochondrial integrity and functions.

**Fig. 6:**
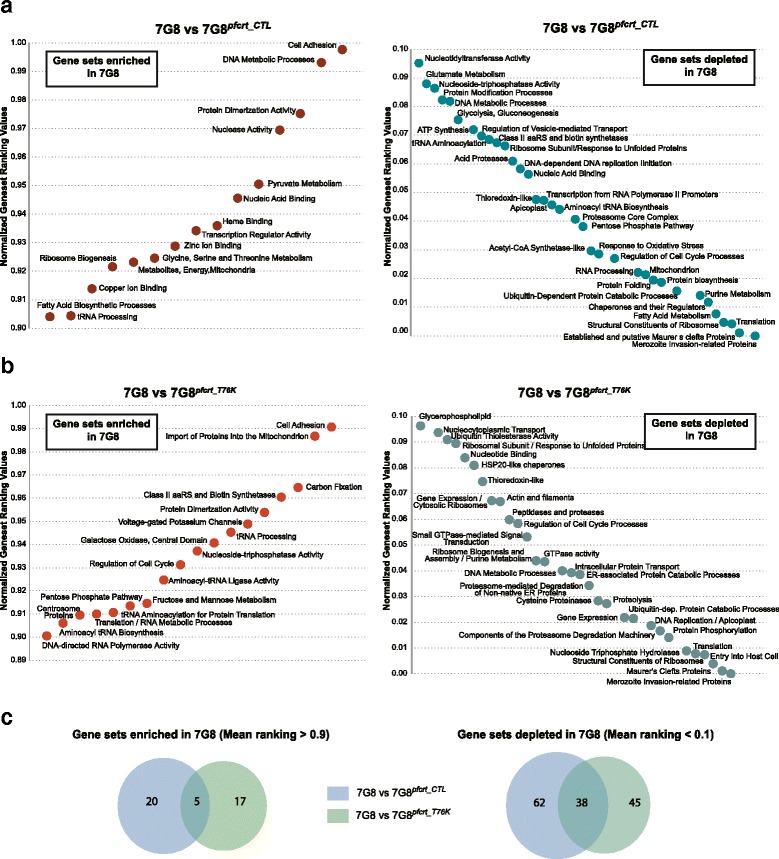
Hypergeometric Analysis of Time Series (HATS) for pairwise comparisons of the transcriptomes in the *pfcrt* data set. As for the *pfmdr1* dataset, we performed HATS analysis by combining FFT to align the time series with a hypergeometric distribution approach based on random permutations to compute an enrichment score for each gene set. Normalization of the enrichment scores was performed using the expression baseline constituted by the background pool of 11 transcriptome data sets. As before, we applied a threshold of normalized gene set rank >0.9 for gene sets we considered positively enriched and normalized gene set rank <0.1 for gene sets considered negatively enriched. The gene sets were built using GO, KEGG, and the Malaria Parasite Metabolic Pathway. **a** Gene sets that are enriched (left, mean ranking values >0.9) or depleted (right, mean ranking values <0.1) in 7G8 in comparison with 7G8^*pfcrt_CTL*^. **b** Gene sets that are enriched (left, mean ranking values >0.9) or depleted (right, mean ranking values <0.1) in 7G8 in comparison to 7G8^*pfcrt_T76K*^. **c** Venn diagrams depicting overlaps of gene sets significantly enriched (left) or depleted (right) in 7G8 for both 7G8 vs. 7G8^*pfcrt_CTL*^ and 7G8 vs. 7G8^*pfcrt_T76K*^ pairwise comparisons

When examining the *pfcrt*-modified transgenic strains, we observed increased expression in several genes encoding putative proteins involved in protein folding, trafficking and turnover (Fig. 5d, e). These include heat shock and DnaJ proteins, which were on average 2-fold more highly expressed in comparison to 7G8 (Additional file 15: Table S9D-F). Increased expression was also observed with *pfhrp3* (PF3D7_1372200), whose product is hypothesized to be involved in hemozoin formation in the parasite digestive vacuole [58]. These observations were confirmed by HATS (Fig. 6), which provided evidence for higher expression of genes in the categories of chaperones and protein catabolism in both 7G8^*pfcrt_CTL*^ and 7G8^*pfcrt_T76K*^ (Fig. 6a, b). Interestingly, there was a substantial overlap between the gene sets depleted in 7G8 in comparison to either 7G8^*pfcrt_CTL*^ or 7G8^*pfcrt_T76K*^ (Fig. 6c). Altogether these results suggest that *pfcrt* transcript levels can influence both parasite-specific processes and general biological pathways irrespective of this transporter’s drug resistance phenotype (see Discussion).

**Fig. 7:**
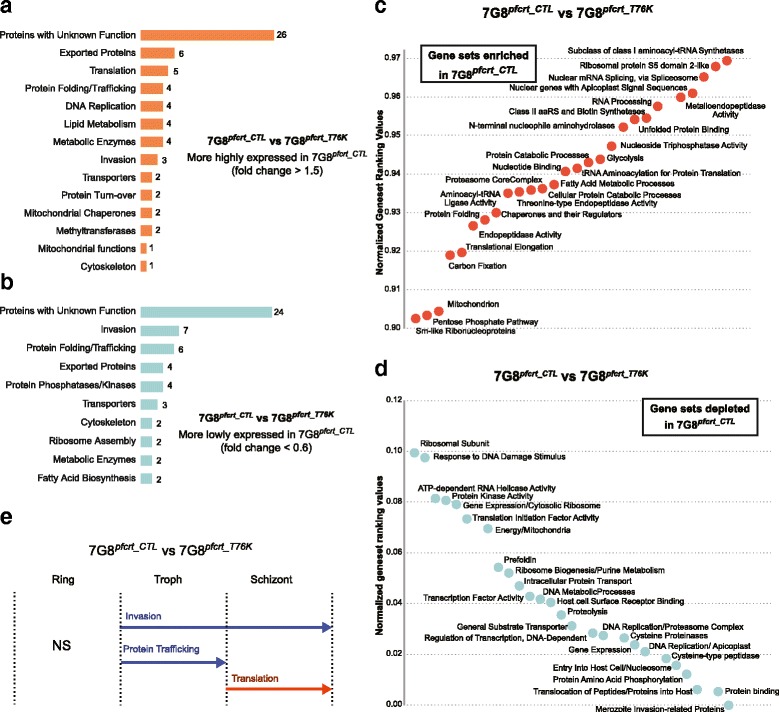
Analysis of the transcriptional differences between 7G8^*pfcrt_CTL*^ and 7G8^*pfcrt_T76K*^. **a** Genes showing higher expression (fold change >1.5 and normalized fold change ≥2 SD above the mean) in the recombinant control 7G8^*pfcrt_CTL*^ in comparison to the recombinant back-mutant strain 7G8^*pfcrt_T76K*^. Gene expression fold changes were calculated as the difference between the areas under the curve generated on the full 384 time point data and were then normalized using a baseline created by the background pool of 11 transcriptomes. **b** Genes showing a lower expression (fold change <0.6 and normalized fold change ≤3 SD below the mean) in the recombinant control 7G8^*pfcrt_CTL*^ in comparison to the recombinant back-mutant strain 7G8^*pfcrt_T76K*^ *.* **c** HATS analysis was used to identify gene sets that are enriched in 7G8^*pfcrt_CTL*^ in comparison to 7G8^*pfcrt_T76K*^ (mean ranking values >0.9). Enrichment scores were normalized using the expression baseline constituted by the background pool of 11 transcriptome data sets. **d** Gene sets that are depleted in 7G8^*pfcrt_CTL*^ in comparison to 7G8^*pfcrt_T76K*^ (mean ranking values <0.1), as identified from HATS analysis. **e** Stage-associated enrichment analysis. Gene sets with significant enrichment (orange) or depletion (blue) in 7G8^*pfcrt_CTL*^ in comparison to 7G8^*pfcrt_T76K*^ for three windows of the parasite IDC corresponding to rings (R, 12–16 h post-invasion), trophozoites (T, 24–28 h post-invasion) and schizonts (S, 32–36 h post-invasion) using stage-enrichment analysis

Other genes whose expression was higher in the transgenic 7G8 strains in comparison to the parental line were mostly involved in parasite invasion and encoded merozoite surface proteins as well as glideosome and rhoptry components (Figs. 5d, e, 6a, b). Interestingly, these effects were accentuated in the back-mutant 7G8^*pfcrt_T76K*^ at both the individual gene as well as gene set levels, during most of the parasite IDC (Fig. 7a-e, Additional files 15 and 16: Table S9 and S10 respectively).

We also compared the transcriptional profiles of 7G8^*pfcrt_T76K*^ and 7G8^*pfcrt_CTL*^ to assess the impact of the CQ resistance phenotype defined by PfCRT K76T. Genes differentially expressed between these lines were enriched for functions associated with DNA/RNA metabolism and protein catabolism (Fig. 7a, b). For instance, multiple genes encoding proteins possibly involved in DNA replication, such as mini-chromosome maintenance proteins (PF3D7_1355100, PF3D7_1417800), DNA helicases (PF3D7_0918600, PF3D7_1227100, PF3D7_1010200), a nucleotide excision repair enzyme (PF3D7_0710400) and the DNA polymerase α subunit (PF3D7_1463300) were ~2-fold more highly transcribed in 7G8^*pfcrt_CTL*^ (Fig. 7a, Additional file 15: Table S9G). Higher expression of some of these genes was observed throughout the IDC as shown by stage-specific analysis (Additional file 18: Table S12). Transcripts involved in RNA processing such as components of the exosome complex (PF3D7_0720000, PF3D7_0209200) showed ~1.5 to 3-fold higher levels in 7G8^*pfcrt_CTL*^, mainly during the ring and trophozoite stages (Additional file 18: Table S12A,C,G). These observations were confirmed by HATS analysis (Fig. 7c, d).

Expression analysis of individual genes and gene sets indicated that genes involved in translation and protein stability, trafficking and turnover, such as those encoding ribosomal subunits and protein chaperones, respectively were also more highly transcribed in the transgenic control line 7G8^*pfcrt_CTL*^ (Fig. 7a, c, Additional file 15: Table S9G). Stage-specific analysis demonstrated that higher expression of some of these genes was detected throughout the entire IDC (Additional file 18: Table S12K). Furthermore, HATS revealed that the categories of amino-acyl-tRNA ligases and synthetases were depleted in the back-mutant strain in comparison to both parental and control lines (Figs. 6b and 7c), although several individual genes within these categories, including some encoding a glutamate-tRNA ligase (PF3D7_1349200 and PF3D7_1357200), displayed higher expression in 7G8^*pfcrt_T76K*^ at either ring or schizont stages (Additional file 18: Table S12B,F). Interestingly, HATS showed that gene sets linked to tRNA aminoacyl biosynthesis were also enriched in 7G8^*pfcrt_CTL*^ in comparison to the parental strain (Fig. 6b). Several tRNA-ligases (PF3D7_0509600, PF3D7_1349200) and ribosyltransferases (PF3D7_1434100) also displayed higher transcript levels in a stage-specific manner (Additional file 16: Table S10B,J), suggesting that translation may also be affected when mutant *pfcrt* expression is lowered (Additional file 19: Figure S7). Pathways linked to proteasome-mediated degradation and ribosome biogenesis were among the multiple genes and gene sets depleted in both control and parental strains in comparison to the back-mutant 7G8^*pfcrt_T76K*^ (Figs. 5e, 6b and 7b, d, Additional files 15: Tables S9K,L). Altogether these observations indicate that the acquisition of drug-resistance mutations in *pfcrt* may impact protein synthesis and turnover pathways.

Expression analysis for individual genes identified several transporters in addition to *pfcrt* that were differentially expressed between the two transgenic strains (Fig. 7a, b, Additional file 15: Table S9I,J), mostly in a stage-specific manner (Additional file 18: Table S12). This finding suggests a possible crosstalk between *pfcrt* and other transporters. For example, ~2-fold higher transcript levels of the ABC transporter PF3D7_0813700 and the folate transporter FT1 (PF3D7_0828600) were detected in 7G8^*pfcrt_CTL*^ (Additional file 18: Table S12A,G), while genes encoding amino acid (PF3D7_0629500) and ABC transporters (PF3D7_1229100 and PF3D7_1352100) were ~1.5-fold more expressed in the back-mutant during either the ring or trophozoite stages (Additional file 18: Table S12D,F).

### Comparison of gene expression patterns across all profiled transcriptomes reveals a transcriptional network that encompasses both *pfcrt* and *pfmdr1*

Our comparative analysis of the transcriptome profiles of the FCB- and 7G8-derived parasite lines demonstrated that mutations and/or altered transcript levels in *pfmdr1* or *pfcrt* impacted the expression of similar gene categories. We therefore assessed whether a transcriptional association between *pfcrt* and *pfmdr1* might account for the similarities we observed between the transcriptome data sets.

**Fig. 8:**
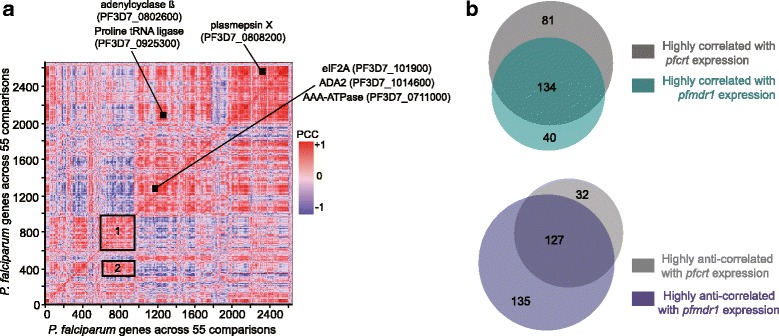
Investigation of *pfcrt* and *pfmdr1* expression networks: Gene expression correlations between all *P. falciparum* genes and *pfcrt*/*pfmdr1*. **a** Cluster heatmap of expression data for *P. falciparum* genes. The hierarchical clustering was generated using Pearson correlation coefficients (PCC) calculated using log_2_-transformed and normalized expression values of 2,600 genes across 55 pairwise comparisons of 11 parasite transcriptome data sets. Heatmaps of the hierarchical clustering show several domains of high correlation between gene pairs, including a large cluster corresponding to strong interactions between plasmepsin X (PF3D7_0808200) and multiple genes involved in cell motility such as myosin (PF3D7_1246400), kinesin-like protein (PF3D7_0724900) and invasion like rhoptry (PF3D7_0414900) or IMC1-related (PF3D7_0304100) proteins. Two smaller clusters (boxes 1 and 2) are shown including one containing both *pfcrt* and *pfmdr1*. **b** Venn diagrams showing the number of genes whose expression highly correlates (PCC >0.7) (top) or anti-correlates (PCC <−0.7) (bottom) with that of both *pfcrt* and *pfmdr1*. PCC values were calculated using log_2_-transformed and normalized expression values of 2600 genes across 110 pairwise comparisons of 11 parasite transcriptome data sets

Using the publicly available transcriptome data sets of the *P. falciparum* strains 3D7, Dd2, and HB3 [46, 48] that we added to our own data sets, we first performed an expression-based hierarchical clustering of the data, using PCC distances between gene pairs computed across all possible pairwise comparisons of transcriptomic profiles. *pfcrt* and *pfmdr1* ranked closely to each other, and formed two distinguishable clusters with high scores for gene pair correlations (Fig. 8a). One of these clusters included a V-type ATPase localized in the parasite digestive vacuole (PF3D7_1354400, PCC ~0.7–0.8), a DnaJ chaperone protein (PF3D7_1307200, PCC ~0.7–0.8) and a transcription factor of the ApiAP2 family (PF3D7_0516800) (Additional file 20: Table S13). The other cluster contained the RNA Polymerase I responsible for rRNA transcription (PF3D7_1134700, PCC 0.8) (Additional file 21: Table S14). Interestingly, the two clusters also contained PF3D7_0823300 that encodes the histone acetyltransferase GCN5 and PF3D7_0303700, a gene involved in lipoamide synthesis, which is in concordance with a recent study reporting the co-expression of *pfcrt* with lipoamide synthesis genes [59]. Altogether these observations suggest an association between *pfmdr1* and *pfcrt* expression and that of genes with specialized functions in the digestive vacuole, in addition to genes having more general functions such as rRNA transcription and protein translation.

To further investigate the transcriptional association between *pfcrt* and *pfmdr1*, we conducted an additional hierarchical clustering of gene expression, this time, by ordering genes based on the level of correlation they displayed with either *pfcrt* or *pfmdr1* expression (Additional file 22: Figure S8). While more than a hundred genes showed a high transcriptional correlation (PCC >0.7) with each gene, we also detected a large number of genes that were strongly anti-correlated (PCC <−0.7) (Additional file 23: Table S15). Interestingly, many genes (~70 %) with strong positive or negative transcriptional correlation with *pfcrt* were additionally strongly correlated with *pfmdr1* expression (Fig. 8b). A dozen of those genes encode transporters, components of the digestive vacuole, or redox enzymes (Table 2). Others were linked to DNA/RNA-dependent processes, translation, and protein trafficking (such as components of the PTEX parasite translocon of exported proteins) (Additional file 24: Table S16A). On the other hand, several genes implicated in either RNA processing or protein catabolism, such as those encoding splicing factors and elements of the proteasome complex, respectively, showed a strong anti-correlation with both *pfcrt* and *pfmdr1* expression (Table 2, Additional file 24: Table S16B). These findings suggest the existence of a transcriptional network that encompasses both genes and evoke a possible functional association between these two drug resistance transporters.

**Table 2:** Table summarizing categories of genes with strong positive (PCC >0.7) and negative correlation (PCC <−0.7) of expression with that of both *pfcrt* and *pfmdr1.* Genes correlated or anticorrelated with pfcrt and pfmdr1 transcript levels

Categories	Gene ID	Description
Positively Correlated with *pfcrt* and *pfmdr*1		
Transporters/Components of the digestive vacuole	PF3D7_0519200	vacuolar ATP synthetase
	PF3D7_0614300	organic anion transporter
	PF3D7_0810200	ABC1 family, putative (ABCK1)
	PF3D7_1132500	amino acid transporter, putative
	PF3D7_1354400	V-type ATPase, putative
	PF3D7_1456800	V-type H()-translocating pyrophosphatase, putative
	PF3D7_1129900	transporter, putative
	PF3D7_1454400	aminopeptidase P
	PF3D7_1126000	threonine--tRNA ligase (ThrRS)
	PF3D7_1332800	translation initiation factor 6, putative
Translation	PF3D7_1367700	alanine--tRNA ligase (AlaRS)
	PF3D7_1445100	histidine--tRNA ligase, putative
	PF3D7_1204300	eukaryotic translation initiation factor 5A (EIF5A)
	PF3D7_0422500	pre-mRNA-splicing helicase BRR2, putative (BRR2)
RNA metabolism	PF3D7_0623900	ribonuclease H2 subunit A, putative
	PF3D7_1421400	DNA-directed RNA polymerase III subunit C, putative
Negatively Correlated with *pfcrt* and *pfmdr1*		
DNA/RNA metabolism	PF3D7_1125300	DNA-dependent RNA polymerase
	PF3D7_0105900	DNA binding protein, putative
	PF3D7_0617800	histone H2A
	PF3D7_1033600	Myb2 protein
	PF3D7_0716100	large ribosomal subunit assembling factor, putative
	PF3D7_0420400	ribosome recycling factor, putative
RNA processing/Translation	PF3D7_0610100	step II splicing factor, putative
	PF3D7_1446900	glutaminyl-peptide cyclotransferase, putative
	PF3D7_0904600	ubiquitin specific protease, putative
Protein Catabolism	PF3D7_0815700	ubiquitin
	PF3D7_0413600	26S proteasome AAA-ATPase subunit RPT3, putative
Transporters	PF3D7_1303500	sodium/hydrogen exchanger, Na, H antiporter
	PF3D7_1352100	ABC transporter, (heavy metal transporter family), putative

## Discussion

Studies have previously defined the malaria parasite transcriptome as hard-wired, capable only of very limited changes at the transcript level in response to specific metabolic challenges [43, 44, 60]. Nevertheless, more recent studies have predicted gene functions and networks using genome-wide transcriptional profiling of *P. falciparum* growth perturbations, and have provided evidence that the parasite can display transcriptional changes even if of low amplitude in response to chemical challenges [61]. In the present study, we have addressed how variant forms of two primary determinants of parasite drug susceptibility can affect the asexual blood stage transcriptome. This focused on a comparative analysis of the transcriptome profiles of *P. falciparum* parasite lines engineered to display distinct levels of expression and/or mutation in the genes coding for the drug resistance transporters PfCRT and PfMDR1. Genome-wide analysis of the transcriptomes was performed both at the gene and gene set levels using computational approaches that identified and excluded transcriptional differences due to stochastic expression. This enabled us to define a transcriptional baseline and detect genes whose expression significantly diverged, implicating them as candidate factors in a parasite transcriptional adaptation to the *pfcrt* and *pfmdr1* changes that were genetically introduced.

Among the ~5,500 coding genes in *P. falciparum* genome, we surveyed the expression changes for a set of 3,000 to 3,500 genes for which the microarray data passed quality inspection across at least 6 of the 8 time points in the series. It is therefore possible that we missed additional genes with differential transcriptional profiles. Despite this limitation, we found that genetic modifications of the loci coding for these two digestive vacuole transporters notably affect the transcription of genes coding for RNA-binding proteins, transcription factors, elements of the translation initiation and elongation machinery, and components of both the spliceosome and proteasome complexes. This observation was particularly striking in the *pfmdr1* data set that included the FCB isogenic strain, FCB^*pfmdr1_3′KD*^. Deamplification of a large region on chromosome 5 that encompassed *pfmdr1* led to the increased expression of genes involved in translation and reduced levels of transcripts implicated in protein catabolism and modifications. This represented in total more than 10 % of the global expression changes and is concordant with expression Quantitative Trait Loci analyses that previously demonstrated an association between the *pfmdr1*-containing amplicon and both post-translational modifications and protein catabolic processes [62]. In agreement with that study [62], we identified a large number of genes involved in proteasome-mediated degradation and protein ubiquitination that display differential expression between FCB and FCB^*pfmdr1_3′KD*^. This suggests that amplification of the *pfmdr1*-containing amplicon can be accompanied by a global reduction of DNA and RNA metabolic activities, and increased protein catabolism.

Our study also identified mechanisms of transcriptional down-regulation across the *pfmdr1* amplicon in both 106/1 (that carries two physical copies) and FCB^*pfmdr1_3′KD*^ (whose second *pfmdr1* copy had been disrupted, and for which the expression of adjacent genes also became downregulated). Earlier studies of Thai field isolates had shown considerable variability in both *pfmdr1* copy number and amplicon size, which would impact expression levels of both *pfmdr1* and its neighboring genes [34]. Our data highlight the need to account for expression when assessing the association between *pfmdr1* copy number and drug susceptibility states, and underscore the potential for additional biological differences resulting from neighboring loci.

Differential expression of genes with a role in fundamental biological processes such as RNA translation, ubiquitin-dependent catabolic processes and protein folding/trafficking was also observed in the *pfcrt* data set, albeit to a lesser extent. Gene sets associated with these pathways showed a greater enrichment in both 7G8-derived transgenic strains that were characterized by a decreased *pfcrt* transcript level, in comparison to the parental 7G8 line. This suggests an association between the degree of *pfcrt* expression and the regulation of cellular protein levels, regardless of PfCRT haplotype.

Intriguingly, levels of *pfcrt* transcripts, irrespective of the transporter polymorphism, also impacted sets of genes implicated in maintaining mitochondrial integrity and function, as indicated by the significant decrease in expression of genes coding for multiple subunits of the translocase complexes of the mitochondria inner membrane [63]. In contrast, the transgenic strains that differ in the nature of the residue at position 76 in PfCRT displayed a differential enrichment of gene sets associated with amino-acyl tRNA synthases and ligases. Several of the genes coding for these elements, which play a central role in RNA translation, were more highly expressed in a stage-specific manner in the transgenic control in comparison to the back-mutant strain. These findings suggest that the impairment of PfCRT transport function through the acquisition of resistance mutations might impact the cellular level of amino acids. This hypothesis is consistent with the observation that PfCRT polymorphisms affect hemoglobin catabolism and that CQ-resistant parasites such as 7G8 are characterized by a phenotype of excessive peptide accumulation, which might in turn impact the reserve of amino acids available for protein synthesis [64]. Additionally, the differential expression of genes encoding putative transporters between the two transgenic strains suggest that PfCRT resistance mutations might more generally affect intracellular transport dynamics, possibly as a secondary effect of the peptide accumulation phenotype.

Interestingly, both *pfmdr1* and *pfcrt* transcriptome data sets displayed differences in the level of expression of genes coding for invasion proteins. Higher transcript levels of rhoptry and merozoite surface proteins, or components of the glideosome were detected in parasite strains with either reduced *pfmdr1* expression such as FCB^*pfmdr1_3′KD*^ and 106/1 or with a PfCRT wild-type haplotype such as the back-mutant strain 7G8^*pfcrt_T76K*^. This, added to the observation that both CQ-sensitive strains 106/1 and 7G8^*pfcrt_T76K*^ had a shorter IDC than the other parasites, suggests an impact of these genetic changes on growth rates and parasite fitness [64]. Interestingly, while 106/1 and FCB have genome sequences that are almost identical [65] (suggesting a common lineage), our data indicate notable differences in both their PfCRT haplotype and their levels of expression from the *pfmdr1* amplicon region. By comparing FCB, FCB^*pfmdr1_3′KD*^ and 106/1, we could disentangle the transcriptional changes due to lower expression/gene copy variation in the chromosome 5 amplicon from the effect of different *pfcrt* genotypes. For instance, 106/1 appears to express higher levels of ribosomal protein genes compared to both FCB strains, suggesting a response to its particular PfCRT haplotype that lacks the CQ resistance-conferring K76T mutation. On the other hand, our data suggest that the transcriptional downregulation of the *pfmdr1*-containing amplicon on chromosome 5 (in 106/1 and FCB^*pfmdr1_3′KD*^) might affect parasite physiology, notably through the reduced expression of genes coding for multiple transporters and components of the parasite digestive vacuole, such as ABC and drug/metabolite transporters and V-type ATPases. Such an observation was particularly striking when performing a comparative analysis of the transcriptomes by stage, demonstrating how such an approach can complement analyses of gene expression averaged across time points.

Our investigations across multiple lines uncovered evidence of co-regulation of *pfcrt* and *pfmdr1,* which both also positively correlated across the genome with a suite of genes coding for transporters, components of the parasite digestive vacuole that include peptidases involved in hemoglobin catabolism, or redox enzymes. Conversely, *pfcrt* and *pfmdr1* transcription anti-correlated with a set of genes encoding proteins involved in RNA processing, including splicing factors. Broader biological processes including transcriptional regulation, translation and post-translational processing contained genes that showed either positive or negative correlations with *pfcrt* and *pfmdr1*. Overall this suggests the existence of an elaborate equilibrium in *P. falciparum* gene expression that maintains optimal states of parasite physiology that extend beyond the digestive vacuole.

Our study builds on the recent work from Siwo and colleagues who constructed *pfcrt* co-expression networks using transcriptional measurements at the early trophozoite stage for the progeny of a genetic cross between CQ-sensitive and CQ-resistant parental strains [59]. Their analysis provided evidence that functional partners of both CQ-resistant and CQ-sensitive *pfcrt* include genes involved in hemoglobin catabolism, which agrees with our own observations. Additionally, the authors attributed divergence in co-expression networks between CQ-resistant and CQ-sensitive *pfcrt* to genes involved in DNA repair and histone acetylation. Interestingly, we found that expression of the histone acetyltransferase GCN5 (PF3D7_0823300) clustered with that of *pfcrt* and *pfmdr1*, further highlighting an association between the expression of these two transporters and general transcriptional processes.

Furthermore, our data extend prior observations of joint contributions of *pfcrt* and *pfmdr1* polymorphisms to drug resistance phenotypes [22, 28, 66], including evidence of linkage disequilibrium, by now providing evidence of a strong correlation between *pfcrt* and *pfmdr1* transcript levels. This has direct implications for drug resistance studies that measure *pfmdr1* copy number as a marker of decreased parasite susceptibility to antimalarial drugs [67] by implying an previously unrecognized role for accompanying changes in *pfcrt*.

## Conclusions

PfCRT and PfMDR1 are two key determinants of *P. falciparum* parasite susceptibility to antimalarial compounds. Both are transporters localized in the intra-erythrocytic digestive vacuole, whose primary functions in addition to carrying antimalarial drugs remain to date unknown. Our transcriptome profiling of various *P. falciparum* strains, whose differences in *pfcrt* or *pdmr1* coding sequence or copy number contribute directly to multidrug resistance, provide insight into how the acquisition of these genetic changes impact parasite physiology beyond the digestive vacuole. These changes include peptide catabolism, digestive vacuole homeostasis, RNA metabolism and protein synthesis. Our findings, based on the development of an analytical method that we term Hypergeometric Analysis of Time Series, also reveal a closely coordinated co-regulation of *pfcrt* and *pfmdr1* transcript levels. Of particular interest now will be to further study how these transcriptional crosstalks affect downstream processes, in particular at the level of the parasite proteome and metabolome.

## Methods

### Parasite culture

All parasite lines described here were propagated under standard conditions [68] in RPMI-1640 medium with L-glutamine, supplemented with 50 mg/l hypoxanthine, 25 mM HEPES, 0.25 % sodium bicarbonate, 0.01 mg/ml gentamicin and 0.5 % Albumax II (Invitrogen, Carlsbad, CA, USA). Parasites were synchronized by two consecutive sorbitol treatments timed 12 h apart for three or more successive generations and allowed to recover for another parasite asexual blood stage cycle before initiating time point samplings. For each time course, cultures were progressively expanded to a volume of 250–300 mL at 3 % hematocrit with 8 to 10 % of tightly synchronous populations of late *P. falciparum* schizonts. Parasites were inoculated in a bioreactor (Applikon, Foster City, CA, USA) in the presence of fresh erythrocytes and allowed to reinvade for the next 2–3 h at high hematocrit (8 %). Gas and temperature conditions were monitored with a Bio Controller unit ADI 1030. The first time point of the series was determined by the peak of invasion, after which cultures were diluted to a final volume of 1,000 mL and 2 % hematocrit. At least 90 % of the parasites were in the early ring stage and in general, cultures reached a parasitemia of 10 to 20 %. Equal parasite samples were harvested every 6 h throughout the 48-hour IDC and immediately centrifuged. Pellets of parasitized erythrocytes were directly stored in Trizol® for subsequent RNA extraction and cDNA synthesis.

### RNA preparation, cDNA synthesis and DNA microarray hybridizations

Total RNA was isolated and synthesis of first strand amino-allyl cDNA was performed as previously reported [69, 70] using 125U of Superscript II reverse transcriptase (Invitrogen, Carlsbad, CA, USA). Each reaction was concentrated on a Zymo DNA clean and concentrator-5 column (Zymo Research, Irvine, CA, USA) and labeled with Cy5 dye (GE Healthcare, Piscataway, NJ, USA). The reference RNA pool consisted of Cy3-coupled cDNA samples prepared from RNAs representing all developmental stages at 6-hour intervals of the IDC of the 3D7 line. Equal amounts of labeled samples from each time point and reference pool were subjected to array hybridization for 16–18 h at 65 °C on 70-mer *P. falciparum* microarray chips that contained 10,416 features and represented all 5,363 coding genes as previously reported [70]. Data were acquired using a GenePix 4000B scanner and analyzed with the GenePix Pro 4 software (Molecular Devices, Sunnyvale, CA, USA).

### Microarray data normalization and quality control

Automatic definition of hybridization spots was refined by manual inspection, and acquired raw data were initially filtered to exclude poor quality spots. The Cy5 and Cy3 intensity channels were separately normalized using the mean difference between the median foreground intensity and the median background intensity of a set of representative probes. This set included probes that contained more than 50 % of the pixels with intensities higher than 2 standard deviations above the background pixel intensity for both Cy5 and Cy3 signals, displayed at least 200 expression units and a regression correlation coefficient greater than or equal to 0.75 [48]. After background subtraction, the log_2_ of the (Cy5/Cy3) intensity ratio was extracted for each probe and gene expression ratios were computed using the median of the probe level values. For genes with values for at least 6 of 8 time points, missing values were imputed using linear model prediction based on PCC values and accounting for mean and standard deviation differences.

Transcriptomes of all lines were reconstituted using the FFT method (*fft()* function in R) to sort genes according to phase, and impute expression levels at 376 unassayed time points, one for each 7.5 min of the 48-hour IDC. To correct for variations in developmental stage speed between parasite lines, dynamic time warping [71] was applied to determine the optimal post-invasion time point (corresponding to the first sampling time point) along the IDC using the PCC between each pair of imputed points. Each parasite line was aligned using the 3D7 transcriptome as reference [46]. The alignments were confirmed by applying PCA. The first two principal components, derived from approximately 3,000 dimensions of expression data, accurately recapitulated the progression of the parasite lines throughout the IDC, while succeeding components focused on differences between parasite lines.

### Microarray data analysis

To assess gene expression level differences between parasite lines, the area under the curve (AUC) of absolute gene expression plots was computed on the full 384 time point data and the fold change was calculated as the difference between AUCs in pairwise comparisons. OPLS-DA (Orthogonal Partial Least Square-Discriminant Analysis) was used to create disjoint PCA models for each parasite line [72] and exploit class-orthogonal variation, thereby permitting the identification of genes expressed out of phase. Both approaches resulted in a similar gene ranking with a Spearman correlation of 0.89. To determine significant variation between parasite lines, a cut-off of >1.5-fold difference in gene expression was assigned.

All values were normalized using a background pool constituted of 11 transcriptome data sets that constituted a baseline for gene expression fold change and gene set enrichment. In addition to that reported in the present study, the pool included two IDC transcriptome data sets generated for Dd2 parasites pressured long-term with pulses of dihydroartemisinin (the 3b1 line) and the non-pressured parent [73], as well as the transcriptome data set previously reported for the 3D7, Dd2 and HB3 strains [46, 48]. This background pool was used to generate 55 pairwise comparisons (or 110, if taking into account analysis in both directions) and create a distribution of fold changes for each analysed gene, which was then used as a normalizing factor. When comparing two lines, only the remaining nine were used to build the fold change distribution. The analysis was conducted using a threshold of two or three standard deviations above this mean fold change. This method, based on a large dataset of pairwise comparisons, enables us to identify even genes for which expression changes are of small amplitudes.

To analyze gene sets, we developed an algorithm that we termed Hypergeometric Analysis of Time Series (HATS). The source code and pseudocode describing HATS are provided in Additional file 25. HATS is based on GSEA [56] and consists in assigning a rank probability to each gene in a given gene set using GSEA and then applying hypergeometric distribution instead of a weighted Kolmogorov-Smirnov-like statistic [56] to evaluate the enrichment score. To this end, 1,000 random permutations of the data were generated, and on each of these permutations we calculated gene rank probabilities *P* for each gene set. These probability values were averaged per gene set across the 1,000 permutations and used as a scaling factor defined as -log_10_(*P*) to generate a corrected enrichment score from that originally computed by GSEA. The false discovery rate (FDR) was estimated by comparing the distributions of the actual enrichment scores to those derived from the random permutations. Noise reduction was achieved by forcing all FDR values to monotonically decrease at the tails. Finally, theses results were then normalized by comparing them to the pool of 11 transcriptome datasets, across which pairwise GSEA comparisons were conducted for all possible combinations. Means and standard deviations of the pool-derived enrichment scores for each gene set were computed and used to generate normalized enrichment scores and remove sets that were stochastically expressed. We applied a cut-off for normalized gene set ranks of 0.1 for negatively enriched gene sets and of 0.9 for positively enriched gene sets.

Results were also analyzed by parasite developmental stages, using time windows corresponding to ring, trophozoite, and schizont stages [74]. Stage-associated enrichment was calculated by averaging gene expression across each developmental stage using the AUC derived from the imputed 384-time point curve and then performing GSEA. As before the enrichment scores were calculated using the hypergeometric distribution and the values were normalized using the background pool of transcriptome datasets.

Gene sets were assembled from gene ontology (GO) annotations [75], the Malaria Parasite Metabolic Pathway [76, 77], KEGG [78] and InterPro [79] databases, and literature searches. A summary of these gene sets is provided in Additional file 24: Table S16. We extracted the GO categories for *P. falciparum* from PlasmoDB (www.plasmoDB.org) such that 1,664 GO categories involving 4,415 genes were included. On average, there were 3.85 categories per gene, while each GO category contained an average of 10.2 genes. There were 16,997 GO category labels applied in total, covering 69.3 % of the genome. On the other hand, 3,888 genes were labeled with an InterPro signature, averaging 3.09 labels per gene. There were 11,997 InterPro labels applied in total, covering 61.0 % of the genome. Finally, the metabolic pathways of *Plasmodium* were extracted from the KEGG database, such that in total 182 metabolic pathways were detailed, averaging 36.7 genes, with pathways ranging from two genes to 342 genes. 2,938 genes were part of at least one pathway, averaging 2.3 pathways per gene. There were 6,671 metabolic pathway assignments applied in total, covering 46.1 % of the genome.

Overall, 7,682 total labels were applied, assigning 5,184 genes to 6,427 gene sets. This accounts for 81.4 % of the genome, each gene being a member on average of 9.2 sets, with the most labeled gene being represented in 45 sets. The portion of the genome that was not represented in these sets included genes having no ascribed functional annotation. Over- or under-representation of a group of functional categories in comparison to all other categories was determined using hypergeometric distribution. Any comparison with a *P-value* < 0.01 was retained.

Hierarchical clustering was performed using PCC values obtained from all pairwise comparisons across all 11 transcriptome data sets using ratio of log_2_-transformed and normalized gene expression values.

### Quantitative real-time PCR and RT-PCR

Real-time PCR was performed in a DNA Engine Opticon 2 detector (Bio-Rad, Hercules, CA, USA) to assess transcript levels and gene copy numbers using the iQ SYBR Green Supermix (Bio-Rad). Parallel amplification reactions were carried out in 96-well plates in 25 μL volume containing 0.3 to 0.5 μM of each forward and reverse primer, with all samples run in triplicates. Measurements were performed on three independent occasions. Primer sequences and real-time PCR conditions are listed in Additional file 27: Table S18.

For quantitative RT-PCR, RNA was treated with DNase I (Ambion) to remove possible traces of contaminating genomic DNA. cDNA was prepared by reverse transcription from 5 μg of total RNA using a mix of oligodT (Invitrogen) and random hexamers (Invitrogen). To assess transcript levels, cDNA templates were diluted 60- to 80-fold. Relative quantification of transcripts was performed using the standard curve method, using 10-fold dilution series of genomic DNA harvested from the parental strains 7G8 or FCB. *cyp87* (PF3D7_0510200, previously PFE0505w, Forward 5′ AAACGGGAGATCCTTCAGGT, Reverse 5′ AAGGACATGGGACAGTGGTT) was used as the reference control gene.

To assess gene copy numbers within *pfmdr1* amplicon, 50 ng of template genomic DNA was used to measure each gene copy number relative to the single-copy *cyp87* or *β-tubulin* (PF3D7_1008700, previously PF10_0084*)* genes. Assays were normalized using standard curves that were generated by 10-fold dilution series of genomic DNA from 3D7, known to have a single copy of *pfmdr1* amplicon. Copy number results were rounded to the nearest integer.

### Availability of supporting data

The data sets supporting the results of this article are available in the GEO (Gene Expression Omnibus) functional genomics data repository at the NCBI, under the accession number GSE75807 (http://www.ncbi.nlm.nih.gov/geo/query/acc.cgi?acc=GSE75807).

## Additional files

Additional file 1: Figure S1. Schema summarizing the parental and transgenic parasite lines used in this study. **(A)** The FCB data set comprises FCB, FCB^*pfmdr1_3′KD*^ and 106/1 (not pictured). FCB^*pfmdr1_3′KD*^ was derived from the multidrug-resistant strain FCB by genetically disrupting one of the two copies of the *pfmdr1* gene, as reported in [26]. pBS: pBluescript plasmid backbone. Δ*mdr*: *pfmdr1* fragment used to disrupt the coding sequence in one of the two endogenous tandem *pfmdr1* loci in FCB, resulting in FCB^*pfmdr1-3′KD*^ (knock-down). **(B)** The 7G8 data set comprises the CQ-resistant parental strain 7G8 (PfCRT haplotype T76), from which two transgenic lines were derived by allelic exchange as previously described [20], namely the control 7G8^*pfcrt_CTL*^ (moderately CQ-resistant, PfCRT haplotype T76) and the back-mutant 7G8^*pfcrt_T76K*^ that is now fully CQ-sensitive (PfCRT haplotype K76). (PDF 347 kb) Additional file 2: Table S1. Validation of the data point imputation. Using the publically available transcriptome datasets for 3D7, Dd2 and HB3 from (Llinas et al. NAR 2007), we validated whether the density of the data in our time series (of eight points, at a 6-hour resolution) was sufficient to impute data to a 7.5 min resolution. For each strain, we selected eight time points evenly spread (6-hrs apart) from which we generated an imputed curve of 384 points. Using Pearson correlation values for gene expression, we then compared each actual hourly time point to the imputed curve to identify the closest matching time point. The imputed percentage column shows where the match occurred in the time series. Actual time points from the original time courses match imputed time points that are fairly evenly spread along the imputation-derived curve. (XLSX 37 kb) Additional file 3: Figure S2. Heatmaps showing alignment of each profiled transcriptome to that of 3D7. FFT was used to impute expression levels from the 8 sampled time points across a data set of 384 time points spanning the 48-hour IDC. This allowed expression level to be extrapolated with close temporal resolution (7.5 min) across the IDC. Dynamic time warping was used to align all 384 points to those of the published 3D7 time series [48, 70], using the PCC score computed between each pair of points. (PDF 2732 kb) Additional file 4: Figure S3. Example of a chromosome map showing differential gene expression in the telomeric regions of chromosome 11 between FCB and both FCB^*pfmdr1_3′KD*^ and 106/1. Expression fold changes for both pairwise comparisons were plotted as a function of the chromosomal coordinates of each gene. Each circle represents a distinct gene. The dashed line delineates the threshold value of a 1.5 fold shift that was interpreted as evidence of transcriptional differences. Note that the (sub)telomeric regions of chromosome 11 are enriched in transcriptionally variable multigene families, notably *var* and *phist* genes. (PDF 129 kb) Additional file 5: Table S2. List of genes within the *pfmdr1*-containing amplicon with associated fold change between FCB and both FCB^*pfmdr1_3′KD*^ and 106/1, as measured by microarrays. (XLSX 11 kb) Additional file 6: Figure S4. Genomic map of the *pfmdr1*-containing amplicon region on chromosome 5 of the FCB strain. Genes listed in Additional file 2: Table S1 are indicated and *pfdmr1* (PF3D7_052300) is boxed in red. (PDF 90 kb) Additional file 7: Table S3. Results of quantitative real time PCR to estimate gene copy numbers associated with gene expression variations between FCB and both FCB_3′KD and 106/1 in the *pfmdr1*-containing amplicon region on chromosome 5. These data suggest dosage compensation for the gene Pf3D7_0521300. (XLSX 57 kb) Additional file 8: Table S4. Values for gene expression fold differences between strains in the 7G8 data set for genes on chromosome 2. The boxed area corresponds to the region on chromosome 2 containing genes with a significant fold change (values <0.6). (XLSX 13 kb) Additional file 9: Figure S5. Venn diagrams depicting the number of genes that were found differentially expressed between FCB and **(A)** FCB^*pfmdr1_3′KD*^ or **(B)** 7G8^*pfcrt_CTL*^ and 7G8^*pfcrt_T76K*^ during one or more developmental stages. Fold changes greater than 1.5 and normalized fold changes two standard deviations above the median value were used for the analyses. (PDF 82 kb) Additional file 10: Table S5. Assessment of a possible time shift in gene expression between parasite lines. We examined the data to assess whether some genes displayed a shifted timing of expression between parasite strains. By comparing peaks of expression for each gene in each pairwise comparison, we determined the number of genes whose peak of expression was shifted of a determined number of hours. 0 corresponds to a shift of less than 1 h, one corresponds to a shift of at least 1 h but less than two hours, etc. Therefore, two genes cannot be more than 24 h apart of each other in either direction. We observed that more than 70 % of all genes peaked within the same 8-hour window in the *pfmdr1* dataset (**Table S5A**) while about 80 % of the genes peaked within the same 5-hour window in the *pfcrt* dataset (**Table S5B**). (XLSX 44 kb) Additional file 11: Table S6 Genes (**A-D**) and gene sets **(E-H**) differentially expressed in the *pfmdr1* data set. For each pairwise comparison, we listed genes with significant differential expression, i.e. with a fold change greater than 1.5 and a normalized fold change two standard deviations above the median values and genes with a fold change lower than 0.6 and a normalized fold change three standard deviations below the median value, which generally represented the quasi-totality of genes with a fold change below 0.6. Normalization was performed using the average fold change computed across a background pool of 11 transcriptome data sets as a scaling factor. This normalization allowed us to identify and filter out most of the hypervariable genes (e.g. *var*, *rifin*, *stevor*, *phist*, and *hyp*). Antigenically variant genes not detected by automatic filtering were manually removed. We listed the gene sets with significant differential expression (based on the mean rank of enrichment score >0.9 (significantly enriched) or <0.1 (significantly impoverished)) that are found in both FCB vs. FCB^*pfmdr1_3′KD*^ and FCB vs. 106/1 pairwise comparisons. (XLSX 65 kb) Additional file 12: Table S7. List of genes with significant differential expression between FCB and FCB^*pfmdr1_3′KD*^ in a stage-specific manner*.* Genes with significantly higher expression displayed absolute fold change values >1.5 and normalized fold changes values two standard deviations above the median normalized fold change value. Fold change for each gene at the designated stage is indicated. No gene appeared to be significantly differently expressed between the two strains during both ring and schizont stages. Genes from antigenic variant families have been removed. (XLSX 71 kb) Additional file 13: Table S8. List of genes with significant differential expression between FCB and 106/1 in a stage-specific manner. Genes with significantly higher expression displayed absolute fold changes >1.5 and normalized fold changes values two standard deviations above the median normalized fold change value. Genes with significantly lower expression had absolute fold change values lower than 0.6 and a normalized fold change three standard deviations below the median value. Fold change for each gene at the designated stage is indicated. (XLSX 66 kb) Additional file 14: Figure S6. Gene set enrichment analysis for the FCB^*pfmdr1_3′KD*^ vs 106/1 pairwise comparison. Gene sets that are significantly **(A)** enriched **(A)** or **(B)** depleted in FCB^*pfmdr1_3′KD*^ in comparison with 106/1. We also performed an analysis of the overlap for **(C)** enriched and **(D)** depleted gene sets between FCB^*pfmdr1_3′KD*^ vs 106/1 and FCB vs 106/1 comparisons. (PDF 162 kb) Additional file 15: Table S9. Genes (**A-F, I-K**) and gene sets (**G-H, L-M**) differentially expressed in the *pfcrt* data set. For each pairwise comparison, 7G8 vs 7G8^*pfcrt_CTL*^, 7G8 vs 7G8^*pfcrt_T76K*^, and 7G8^*pfcrt_CTL*^ vs 7G8^*pfcrt_T76K*^, we listed genes with significant differential expression, i.e. with a fold change greater than 1.5 and a normalized fold change two standard deviations above the median values. We listed the gene sets significantly enriched and depleted in 7G8 in comparison with both 7G8^*pfcrt_CTL*^ and 7G8^*pfcrt_T76K*^, and in 7G8 and 7G8^*pfcrt_CTL*^ in comparison to 7G8^*pfcrt_T76K*^. (XLSX 48 kb) Additional file 16: Table S10. List of genes with significant differential expression between 7G8 and 7G8^*pfcrt_CTL*^ in a stage-specific manner. Genes with significantly higher expression displayed absolute fold changes >1.5 and normalized fold changes values two standard deviations above the median normalized fold change value. Genes with significantly lower expression had absolute fold change values lower than 0.6 and a normalized fold change three standard deviations below the median value. Fold change for each gene at the designated stage is indicated. (XLSX 55 kb) Additional file 17: Table S11. Genes with significant differential expression between 7G8 and 7G8^*pfcrt_T76K*^ in a stage-specific manner. Genes with significantly higher expression displayed absolute fold changes >1.5 and normalized fold changes values two standard deviations above the median normalized fold change value. Genes with significantly lower expression had absolute fold change values lower than 0.6 and a normalized fold change three standard deviations below the median value. Fold change for each gene at the designated stage is indicated. (XLSX 59 kb) Additional file 18: Table S12. Genes with significant differential expression between 7G8^*pfcrt_CTL*^ and 7G8^*pfcrt_T76K*^ in a stage-specific manner. Genes with significantly higher expression displayed absolute fold changes >1.5 and normalized fold changes values two standard deviations above the median normalized fold change value. Genes with significantly lower expression had absolute fold change values lower than 0.6 and a normalized fold change three standard deviations below the median value. Fold change for each gene at the designated stage is indicated. (XLSX 58 kb) Additional file 19: Figure S7. Gene sets with significant enrichment (orange) or depletion (blue) in 7G8 in comparison to 7G8^*pfcrt_CTL*^ for three windows of the parasite IDC corresponding to rings (R, 12–16 h post-invasion), trophozoites (T, 24–28 h post-invasion) and schizonts (S, 32–36 h post-invasion). (PDF 110 kb) Additional file 20: Table S13. Hierarchical clustering, which was performed using Pearson correlation coefficients between ~2600 gene pairs across 55 pairwise comparisons of 11 parasite transcriptome data sets, including FCB and 7G8 data sets. Cluster 1 corresponding to gene pairs with high Pearson correlation (PCC >0.7), containing *pfmdr1 and pfcrt* as well as a transcription factor of the ApiAP2 family PF3D7_0516800. (XLSX 42 kb) Additional file 21: Table S14. Cluster 2 from the Hierarchical clustering, corresponds to another group of gene pairs with high Pearson correlation (PCC >0.7) and includes *pfmdr1* and *pfcrt*. (XLSX 46 kb) Additional file 22: Figure S8. Cluster heatmap of gene expression data for *pfcrt*/*pfmdr1* and all other genes. The hierarchical clustering was generated using PCC values calculated using log_2_-transformed and normalized expression values of 2,600 genes across 110 pairwise comparisons of 11 parasite transcriptome data sets, corresponding to all possible combinations in both directions (i.e. A vs. B and B vs. A). Squares indicate areas containing genes that are all strongly correlated or strongly anti-correlated with the expression of both *pfcrt* and *pfmdr1*. (PDF 499 kb) Additional file 23: Table S15. Gene expression correlation between *P. falciparum* genes and either *pfcrt* or *pfmdr1*. Pearson correlation coefficients were calculated between *pfcrt*/*pfmdr1* expression values and those of other 2,600 *P. falciparum* genes across 110 pairwise comparisons. These correspond to bi-directional comparisons between 11 parasite transcriptome data sets, including FCB and 7G8 data sets. **(A)** List of genes whose expression is highly correlated (PCC >0.7) to that of *pfcrt*. **(B)** List of genes whose expression is highly anti-correlated (PCC <−0.7) to that of *pfcrt*. **(C)** List of genes whose expression is highly correlated (PCC >0.7) to that of *pfmdr1.* **(D)** List of genes whose expression is highly anti-correlated (PCC <−0.7) to that of *pfmdr1*. Corresponding Spearman correlations are also indicated. (XLSX 87 kb) Additional file 24: Table S16. Identification of genes whose expression is strongly correlated to that of both *pfcrt* and *pfmr1*. PCC were estimated from 110 pairwise comparisons of 11 transcriptome data sets. **(A)** List of genes organized by functional categories whose expression is highly correlated (PCC >0.7) to that of both *pfcrt* and *pfmdr1*. *Indicates genes with confidence, as found when using PCC computed from 55 unidirectional pairwise comparisons of the 11 transcriptome data sets. **(B)** List of genes organized by functional categories whose expression is highly anti-correlated (PCC<−0.7) to that of both *pfcrt* and *pfmdr1*. *Indicates genes with confidence, as found when using PCC computed from 55 unidirectional pairwise comparisons of the 11 transcriptome data sets. (XLSX 42 kb) Additional File 25:
**Zipped file folder containing code and pseudocode for HATS analysis.** These files can be downloaded from http://www.fidock.org/data/fidock_source_code.zip. (DOCX 32 kb) Additional File 26: Table S17. Table summarizing all the gene sets, extracted from various databases or built by our group, that were used in this study. (XLSX 688 kb) Additional file 27: Table S18. List of the primers used for the qPCR and qRT-PCR reactions. (XLSX 31 kb)

## Abbreviations

AUC: area under the curve
ACTs: artemisinin-based combination therapies
CQ: chloroquine
IDC: intra-erythrocytic developmental cycle
FDR: false discovery rate
FFT: Fast Fourier Transform
GO: gene ontology
GSEA: Gene Set Enrichment Analysis
HATS: Hypergeometric Analysis of Time Series
OPLS-DA: Orthogonal Partial Least Square-Discriminant Analysis
PCA: principal component analysis
PCC: Pearson correlation coefficient
PfCRT: *Plasmodium falciparum* Chloroquine Resistance Transporter
PfMDR1: *Plasmodium falciparum* Multidrug Resistance 1
SD: standard deviation
