# Supplementary figures and images for: Genome-wide transcriptome profiling reveals functional networks involving the Plasmodium falciparum drug resistance transporters PfCRT and PfMDR1
Source: BMC Genomics. 2015 Dec 21;16:1090. doi: 10.1186/s12864-015-2320-8 (PMC4687325; doi:10.1186/s12864-015-2320-8)

# Figure S1

## A

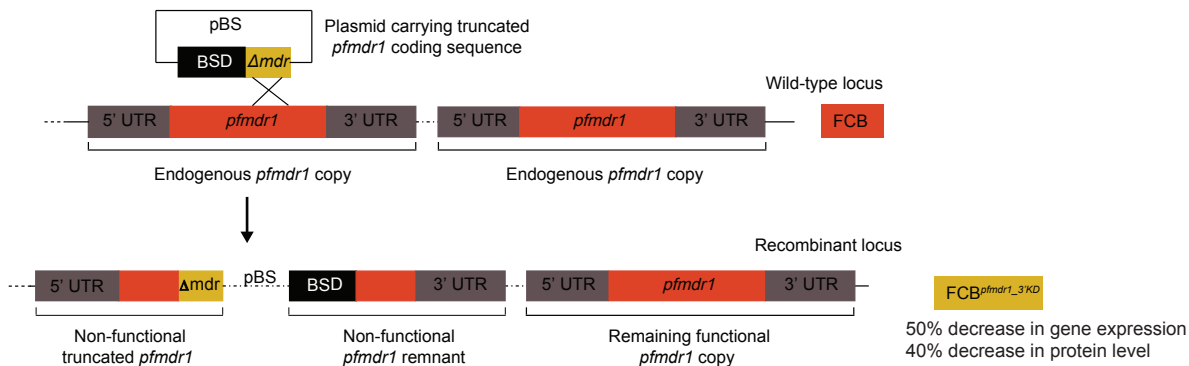

## B

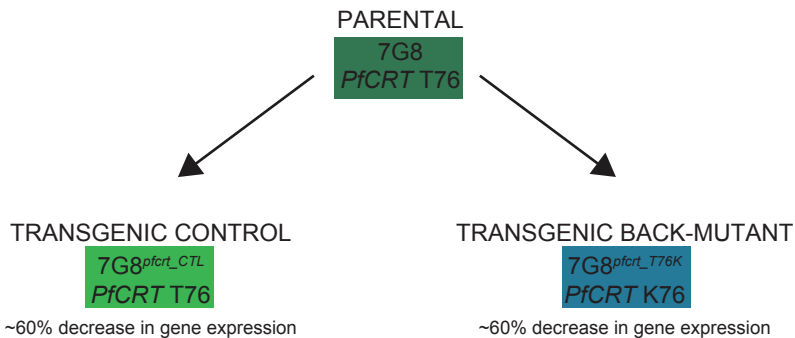

Supplement: Additional file 1: Figure S1. — Schema summarizing the parental and transgenic parasite lines used in this study. (A) The FCB data set comprises FCB, FCBpfmdr1_3′KD and 106/1 (not pictured). FCBpfmdr1_3′KD was derived from the multidrug-resistant strain FCB by genetically disrupting one of the two copies of the pfmdr1 gene, as reported in [26]. pBS: pBluescript plasmid backbone. Δmdr: pfmdr1 fragment used to disrupt the coding sequence in one of the two endogenous tandem pfmdr1 loci in FCB, resulting in FCBpfmdr1-3′KD (knock-down). (B) The 7G8 data set comprises the CQ-resistant parental strain 7G8 (PfCRT haplotype T76), from which two transgenic lines were derived by allelic exchange as previously described [20], namely the control 7G8pfcrt_CTL (moderately CQ-resistant, PfCRT haplotype T76) and the back-mutant 7G8pfcrt_T76K that is now fully CQ-sensitive (PfCRT haplotype K76). (PDF 347 kb) [file 12864_2015_2320_MOESM1_ESM.pdf]

Figure S2

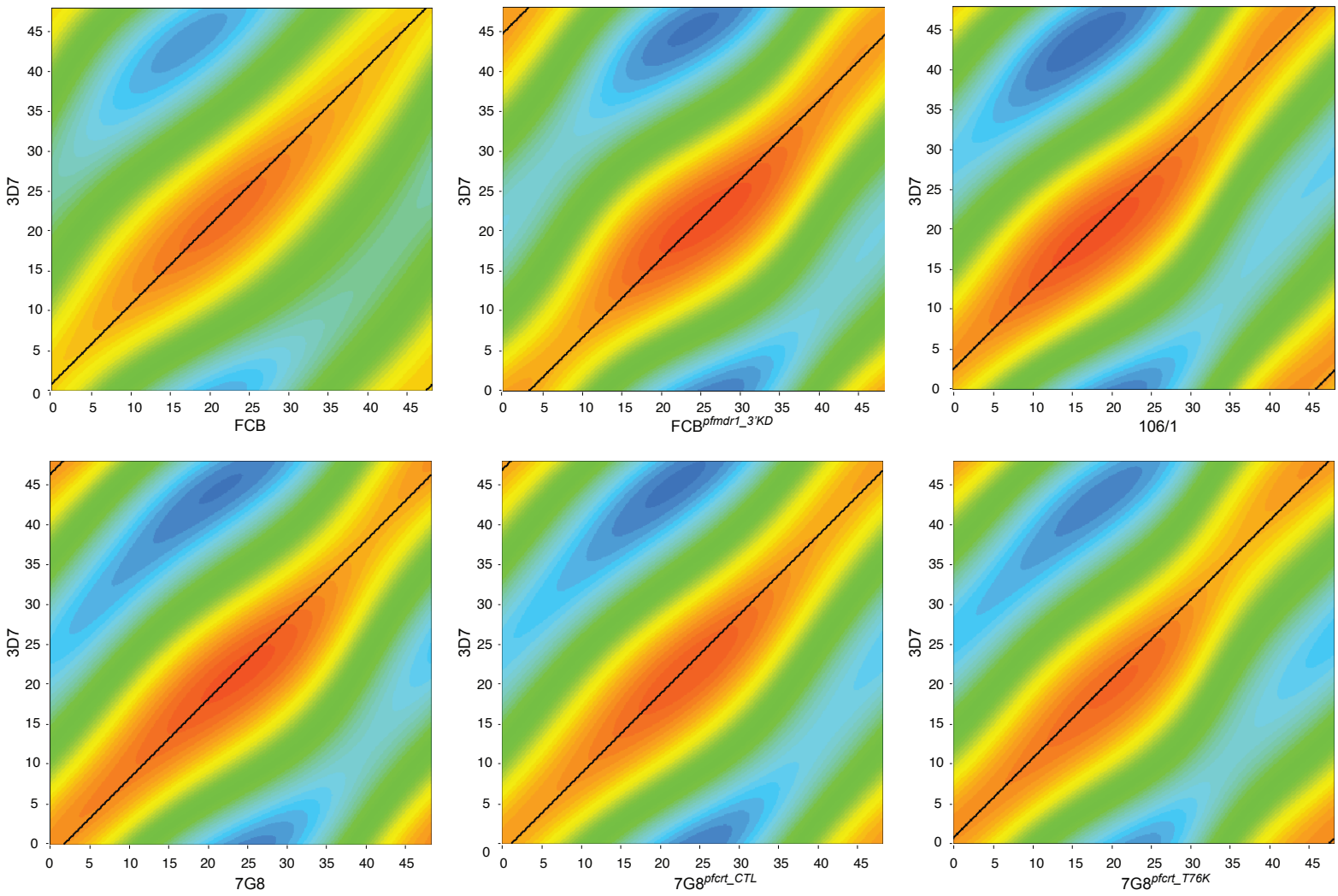

Supplement: Additional file 3: Figure S2. — Heatmaps showing alignment of each profiled transcriptome to that of 3D7. FFT was used to impute expression levels from the 8 sampled time points across a data set of 384 time points spanning the 48-hour IDC. This allowed expression level to be extrapolated with close temporal resolution (7.5 min) across the IDC. Dynamic time warping was used to align all 384 points to those of the published 3D7 time series [48, 70], using the PCC score computed between each pair of points. (PDF 2732 kb) [file 12864_2015_2320_MOESM3_ESM.pdf]

# Figure S3

Gene Expression Fold Change on Chromosome 11

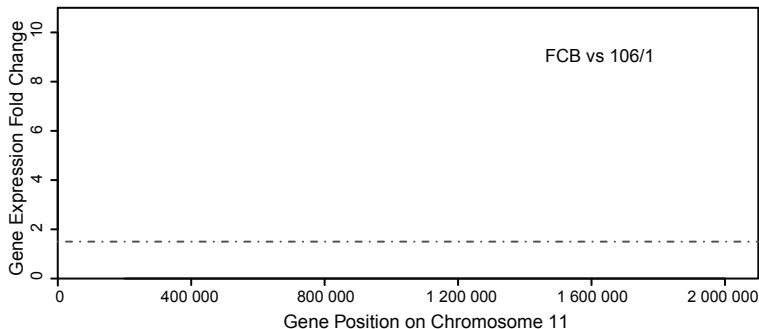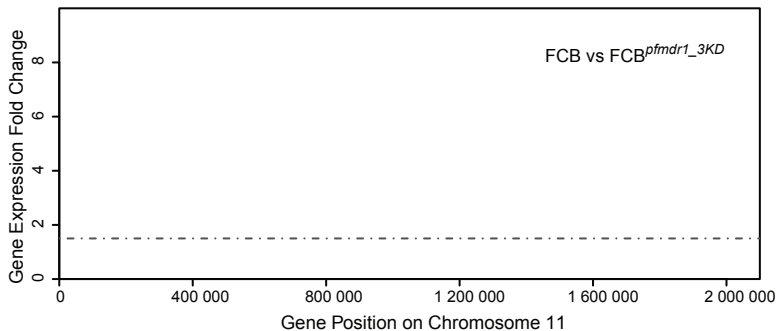

Supplement: Additional file 4: Figure S3. — Example of a chromosome map showing differential gene expression in the telomeric regions of chromosome 11 between FCB and both FCBpfmdr1_3′KD and 106/1. Expression fold changes for both pairwise comparisons were plotted as a function of the chromosomal coordinates of each gene. Each circle represents a distinct gene. The dashed line delineates the threshold value of a 1.5 fold shift that was interpreted as evidence of transcriptional differences. Note that the (sub)telomeric regions of chromosome 11 are enriched in transcriptionally variable multigene families, notably var and phist genes. (PDF 129 kb) [file 12864_2015_2320_MOESM4_ESM.pdf]

Figure S4

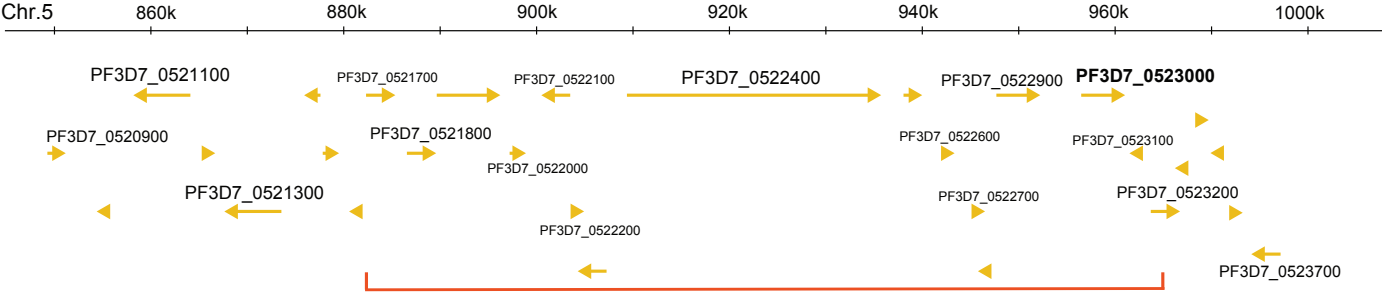

Supplement: Additional file 6: Figure S4. — Genomic map of the pfmdr1-containing amplicon region on chromosome 5 of the FCB strain. Genes listed in Additional file 2: Table S1 are indicated and pfdmr1 (PF3D7_052300) is boxed in red. (PDF 90 kb) [file 12864_2015_2320_MOESM6_ESM.pdf]

# Figure S5

## A

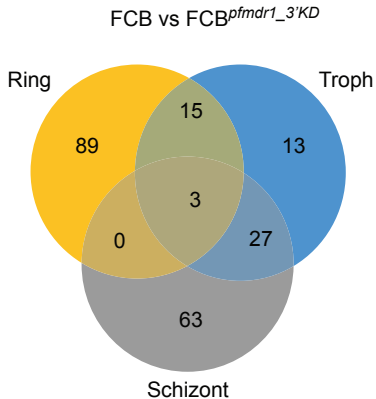

## B

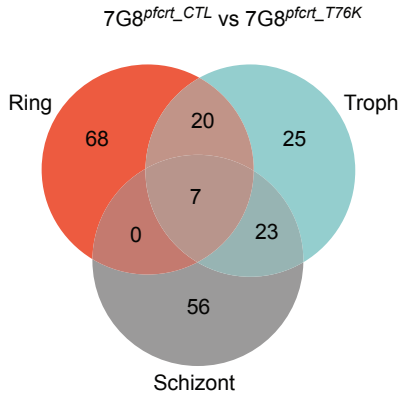

Supplement: Additional file 9: Figure S5. — Venn diagrams depicting the number of genes that were found differentially expressed between FCB and (A) FCBpfmdr1_3′KD or (B) 7G8pfcrt_CTL and 7G8pfcrt_T76K during one or more developmental stages. Fold changes greater than 1.5 and normalized fold changes two standard deviations above the median value were used for the analyses. (PDF 82 kb) [file 12864_2015_2320_MOESM9_ESM.pdf]

# Figure S6

**A**

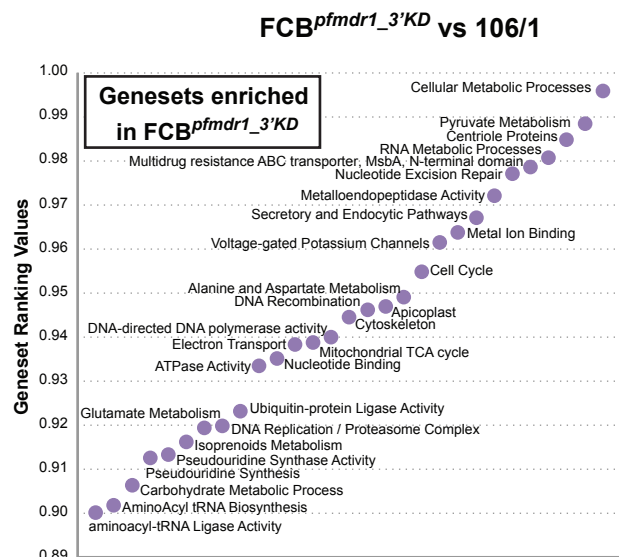

**B**

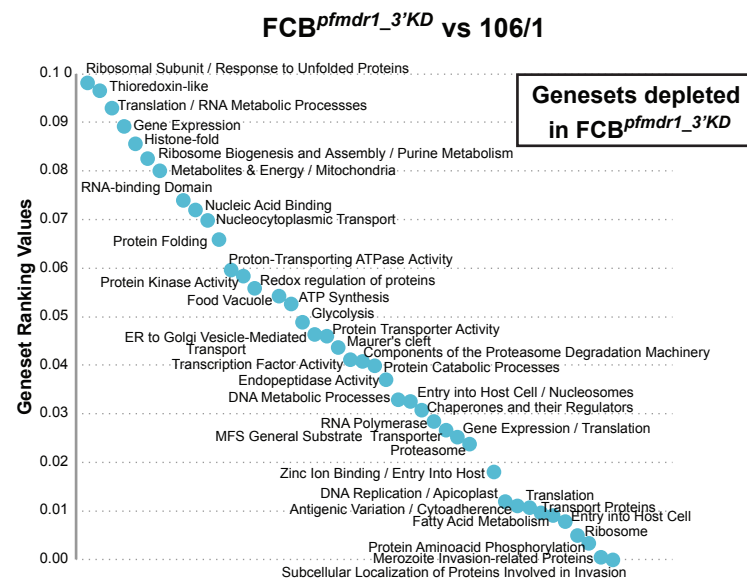

**C**

**Enriched Genesets (Mean ranking > 0.9)**

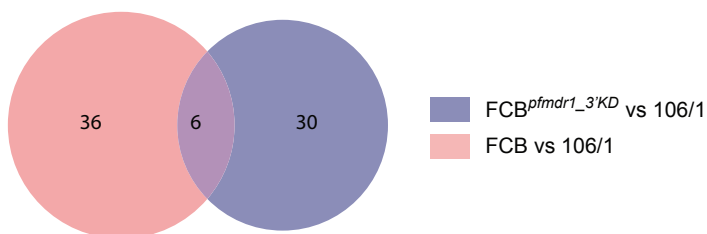

**D**

**Depleted Genesets (Mean ranking < 0.1)**

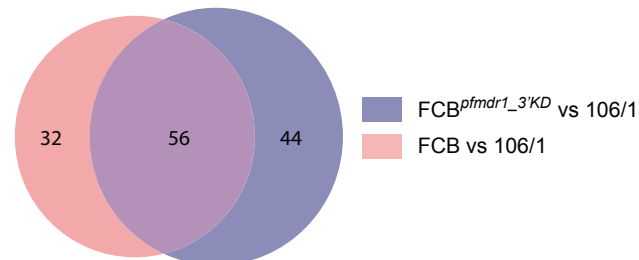

Supplement: Additional file 14: Figure S6. — Gene set enrichment analysis for the FCBpfmdr1_3′KD vs 106/1 pairwise comparison. Gene sets that are significantly (A) enriched (A) or (B) depleted in FCBpfmdr1_3′KD in comparison with 106/1. We also performed an analysis of the overlap for (C) enriched and (D) depleted gene sets between FCBpfmdr1_3′KD vs 106/1 and FCB vs 106/1 comparisons. (PDF 162 kb) [file 12864_2015_2320_MOESM14_ESM.pdf]

# Figure S7

7G8 vs 7G8<sup>pfcr1-CTL</sup>

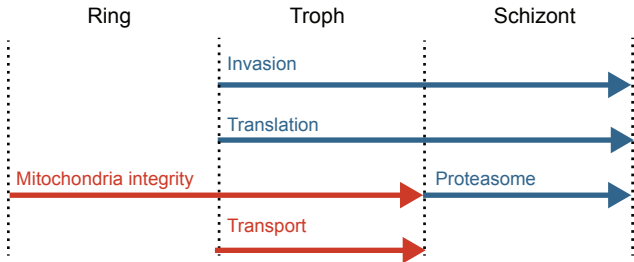

Supplement: Additional file 19: Figure S7. — Gene sets with significant enrichment (orange) or depletion (blue) in 7G8 in comparison to 7G8pfcrt_CTL for three windows of the parasite IDC corresponding to rings (R, 12–16 h post-invasion), trophozoites (T, 24–28 h post-invasion) and schizonts (S, 32–36 h post-invasion). (PDF 110 kb) [file 12864_2015_2320_MOESM19_ESM.pdf]

# Figure S8

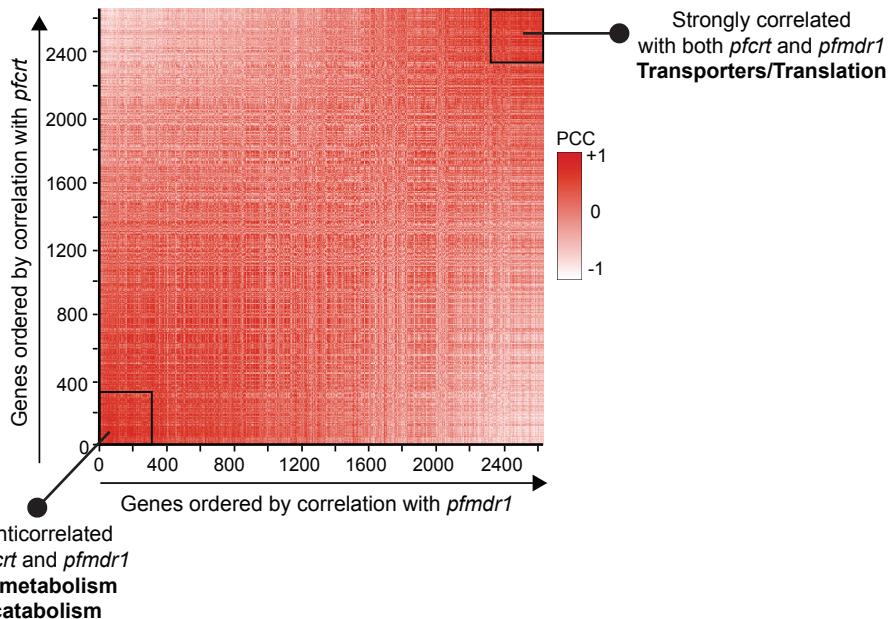

Supplement: Additional file 22: Figure S8. — Cluster heatmap of gene expression data for pfcrt/pfmdr1 and all other genes. The hierarchical clustering was generated using PCC values calculated using log2-transformed and normalized expression values of 2,600 genes across 110 pairwise comparisons of 11 parasite transcriptome data sets, corresponding to all possible combinations in both directions (i.e. A vs. B and B vs. A). Squares indicate areas containing genes that are all strongly correlated or strongly anti-correlated with the expression of both pfcrt and pfmdr1. (PDF 499 kb) [file 12864_2015_2320_MOESM22_ESM.pdf]
