# Supplementary material for: Genome-wide transcriptome profiling reveals functional networks involving the Plasmodium falciparum drug resistance transporters PfCRT and PfMDR1
Source: BMC Genomics. 2015 Dec 21;16:1090. doi: 10.1186/s12864-015-2320-8 (PMC4687325; doi:10.1186/s12864-015-2320-8)
Supplement: Additional File 25: — Zipped file folder containing code and pseudocode for HATS analysis. These files can be downloaded from http://www.fidock.org/data/fidock_source_code.zip. (DOCX 32 kb) [file 12864_2015_2320_MOESM25_ESM.docx]

The files containing codes and pseudocodes for HATS analysis can be downloaded from <http://www.fidock.org/data/fidock_source_code.zip>.
